# Calculation of a Primary Immunodeficiency “Risk Vital Sign” via Population-Wide Analysis of Claims Data to Aid in Clinical Decision Support

**DOI:** 10.3389/fped.2019.00070

**Published:** 2019-03-18

**Authors:** Nicholas L. Rider, Di Miao, Margaret Dodds, Vicki Modell, Fred Modell, Jessica Quinn, Heidi Schwarzwald, Jordan S. Orange

**Affiliations:** ^1^Section of Immunology-Allergy, Rheumatology and Retrovirology, Department of Pediatrics, Baylor College of Medicine, Texas Children's Hospital, Houston, TX, United States; ^2^Department of Pediatrics, Texas Children's Health Plan, Baylor College of Medicine, Texas Children's Hospital, Houston, TX, United States; ^3^The Jeffrey Modell Foundation, New York, NY, United States; ^4^Department of Pediatrics, Columbia University Vagelos College of Physicians and Surgeons, New York-Presbyterian/Morgan Stanley Children's Hospital, New York, NY, United States

**Keywords:** primary immumunodeficiencies, biomedical informatics, public health, biomedical informatics and mathematics, big data and analytics

## Abstract

**Background:** Early diagnosis of primary immunodeficiency disease leads to reductions in illness and decreased healthcare costs. Analysis of electronic health record data may allow for identification of persons at risk of host-defense impairments from within the general population. Our hypothesis was that coded infection history would inform individual risk of disease and ultimately lead to diagnosis.

**Methods:** In this study we assessed individual risk for primary immunodeficiency by analyzing diagnostic codes and pharmacy records from members (*n* = 185,892) of a large pediatric health network. Relevant infection-associated diagnostic codes were weighted and enumerated for individual members allowing for risk score calculations (“Risk Vital Sign”). At-risk individuals underwent further assessment by chart review and re-analysis of diagnostic codes 12 months later.

**Results:** Of the original cohort, 2188 (1.2%) individuals were identified as medium-high-risk for having a primary immunodeficiency. This group included 41 subjects who were ultimately diagnosed with primary immunodeficiency. An additional 57 medium-high risk patients had coded diagnoses worthy of referral.

**Conclusions:** Population-wide informatics approaches can facilitate disease detection and improve outcomes. Early identification of the 98 patients with confirmed or suspected primary immunodeficiency described here could represent an annual cost savings of up to $7.7 million US Dollars.

## Introduction

Biomedical informatics techniques offer potential for understanding large data sets and harnessing vast networks toward optimizing diagnostic accuracy and patient outcomes (1–3). In fact, the National Library of Medicine and the National Institutes of Health recently released statements calling for novel methods of data-driven research to advance biomedical discovery and optimize healthcare (4, 5). Tools such as natural language processing, machine learning, and computer-aided diagnostic algorithms can mine large data sets such as electronic health records (EHRs) and data warehouses to facilitate quality and precise care (6–10). These tools can then inform clinical decision support (CDS) systems facilitating use of the EHR for direct patient care (11–14). In the field of Clinical Immunology, computational algorithms show promise for diagnosis and tracking of patients with Primary Immunodeficiency Diseases (PIs) (15, 16). Presently, informatics tools are not yet widely implemented; however, they offer important potential in allowing for expedited diagnosis and ongoing quality outcome tracking in this vulnerable patient population.

Primary immunodeficiencies represent a heterogeneous group of 354 distinct disorders which interrupt normal host-defense mechanisms (17). Presently, newborn screening is available for T-cell deficiencies; however, more common forms of PI often go undetected, leading to adverse patient outcomes and excessive costs (18). To both address concerns about delays in diagnosis for PI patients and track optimal outcomes, the Software for Primary Immunodeficiency Recognition Intervention and Tracking (SPIRIT) Analyzer was created. This customized computational tool leverages over 350 weighted International Classification of Disease 9 and 10 (ICD-9, ICD-10) and pharmacy codes paired to rational clinical metrics of impaired immune function to classify individuals as low, medium or high risk of having a PI (15). In a previous report, pilot testing on over 2 million patients from within the adjudicated IMS Health LifeLink Health Plan Claims Database, the Analyzer showed promise for risk screening and detection of known PI patients. That study investigated the Analyzer's performance, noting a specificity of 100% and sensitivity of 63% (15).

To further understand performance and utility of the algorithm in a large and ethnically diverse population, we examined over 9 million ICD9/ICD10 codes and pharmacy codes for 185,892 patients from within the Texas Childrens' Health Plan. We hypothesized that weighted diagnosis codes and pharmacy records may inform individual risk for PI across a population and could therefore be utilized as a clinical decision aid during routine patient care. This is important because the presentation of PI is variable and recognition of the disease entity can be challenging (19). Also, among patients with immune deficiency, annual disease-related care exceeds $100,000 and post-diagnosis cost savings are calculated to be $78,166 (USD) even when cost of therapy is accounted for (15, 16, 20). For these reasons, early detection of PI is important and big data driven methods of detection can be important. Our study suggests value in mining large and accessible data sets such as medical and pharmacy claims for PI risk assessment and facilitation of CDS.

## Materials and Methods

This study was approved by the Baylor College of Medicine Institutional Review Board under study H-38501. We obtained de-identified health claims data on 185,892 patients (ages birth-−64 years) who were enrolled in the Texas Children's Health Plan between January 1, 2016 and June 30, 2016. The cohort represented a wide cross-section of a major metropolitan area which encompassed urban, suburban and rural areas. We selected an entire 6-month population of Health Plan members without bias. The only inclusion criteria was Health Plan membership. The only exclusion criteria was having previously received a coded diagnosis for a PI.

To better understand burden of PI in our local patient population, we surveyed all health and pharmacy claims for each of the 185,892 individuals over the January 1, 2016 to June 30, 2016 timeframe. Individual risk of PI was calculated by our algorithm (freely available at www.info4pi.org/town-hall/spirit) as previously described by enumerating the health codes deemed to be informative about risk of underlying host-defense impairment (15). Each of the 350+ informative codes was assigned a weight of 1, 2, or 3 based upon perceived severity of infection as determined by a team of clinical immunologists who routinely care for patients with PI (Supplemental Tables 1, 2). Infection severity was weighted in such a way that generally mild infections (e.g., tonsillitis, acute sinusitis, otitis media) yield 1 point; whereas, severe infections (e.g., pneumocystis pneumonia, pneumococcal sepsis and bacterial meningitis) yield 3 points. Intermediate infections add 2 points to the score. Enumeration of points for a given patient results in their risk score (“Risk Vital Sign”) based upon the health and pharmacy codes entered over the timeframe of interest.

Categorization of individual patient risk was based upon the patient's score. Patients receiving >10 points were considered “high risk,” patients receiving 8–10 points were classified as “medium risk” and patients with scores between 1 and 7 were deemed “low risk.” To allow for routine childhood infections, no points are generated for ear infections without having at least 4 episodes per year or for sinus infections without having at least 2 episodes per year (i.e., 1 point for each episode of otitis media for the 5th and subsequent infection; 1 point for each episode of sinus infection for the 3rd and subsequent.). Pharmacy claims contributed to the risk score only if a patient required 60 or more days of continuous antibiotic therapy. In that event, the patient was given three points for each 60 day course. Following generation of risk scores for our cohort, patients with scores of 8 or greater were deemed to be medium-high risk (MHR).

**Figure 1:**
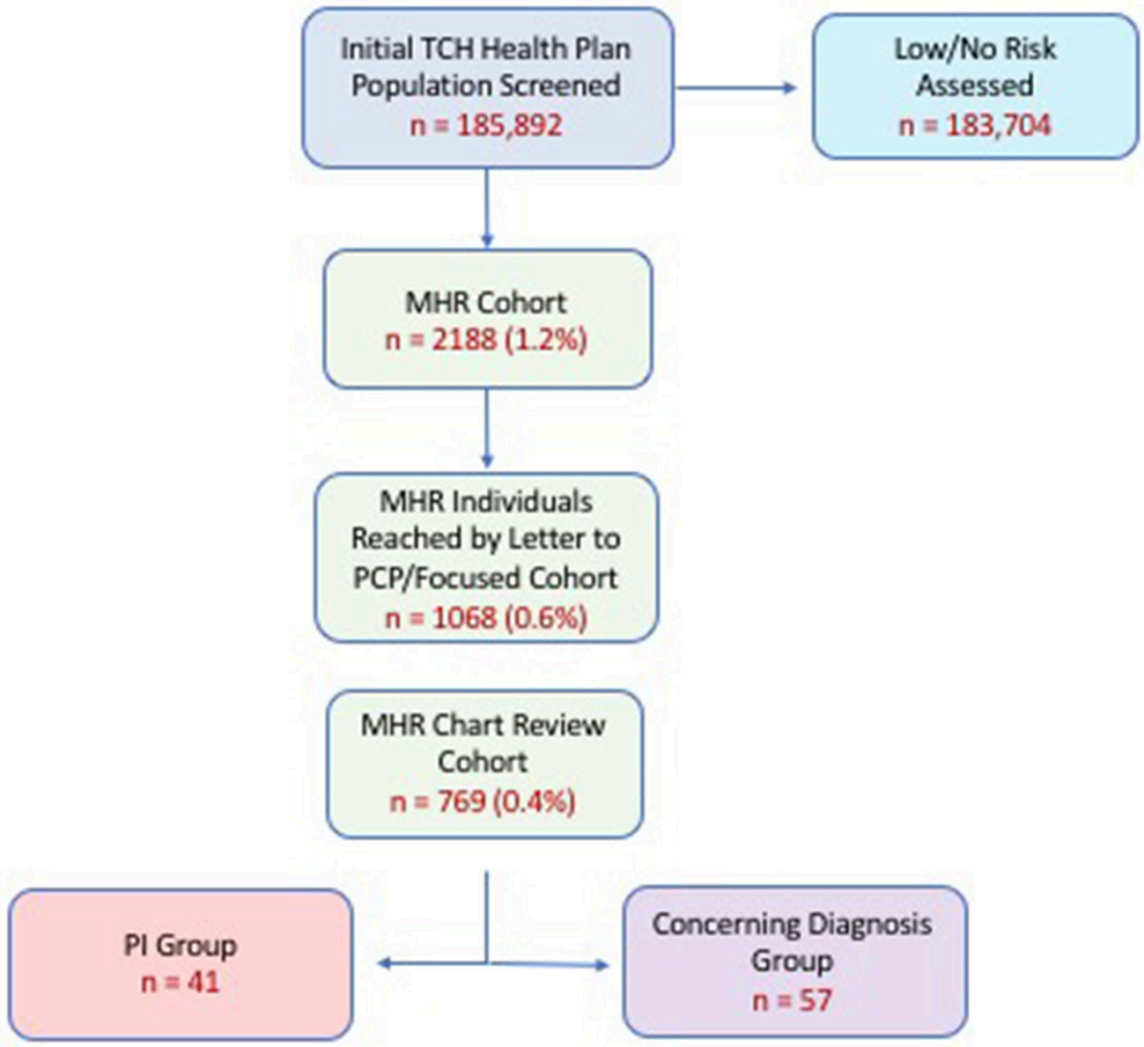
Cohort analysis by stepwise progression. Percentages in parentheses represent proportion of original cohort (i.e., % of 185,892). PI (Primary Immunodeficiency) Group refers to those individuals who were coded a PI-related diagnosis upon re-analysis 12 months after initial screening. Concerning Diagnosis Cohort refers to individuals who were given a diagnosis warranting further evaluation by a clinical immunologist. Attrition shown here related to individuals who left the health plan and/or sought care outside of our health system. (MHR, Medium-High Risk).

Following claim analysis, letters were sent to pediatricians of MHR patients to report our findings and ask about provider's open-ended perception of risk of PI. This was considered to be a “targeted intervention.” Of the total MHR group a focused MHR group (MHR members remaining in the Health Plan 12 months later) of patients was re-analyzed for outcomes (i.e., diagnostic and pharmacy claims, clinic visits, labs, referral, additional diagnoses) over the timeframe of July 1, 2016-June 30, 2017. The purpose of re-analysis was to determine if any MHR patients had been given a PI diagnosis in the 12 months following initial screening assessment and primary care physician notification. All MHR individuals remaining in the health plan at follow up, with available EHRs (MHR Chart Review Cohort; n = 769) underwent manual chart review for more detailed clinical assessment about health outcomes (Figure 1).

## Results

Characteristics of the Health Plan's main cohort and MHR cohort are shown in Table 1. We found approximately 1% (2,188 patients) of the original cohort, which was assessed to be medium-high risk for having a PI. A breakdown of the risk scores for the MHR Cohort is shown in Supplemental Table 2. Of the original MHR group, 1,068 (0.6%) MHR patients remained in the health plan for a targeted intervention (letter to physician) and subsequent re-assessment one year later. From this focused MHR group that received the targeted intervention, 41 (0.02% of Main Cohort; 3.8% of focused MHR Cohort) were ultimately coded as having a PI in the 12 months after our original assessment. Another 57 patients (0.03% of Main Cohort; 5.3% of focused MHR Cohort) had medical conditions coded which were concerning for an underlying PI (Figure 1). The focused MHR group was similar to the general population except that it had a greater percentage of individuals 5 years of age or less (41% in MHR vs. 18.9% in Main).

**Table 1:** Cohort demographics.

**Ethnicity**	**Main cohort (no)**	**%**	**Focused MHR cohort (no)**	**%**
Hispanic	111413	59.9	636	59.5
No ethnicity noted	16227	8.7	146	13.7
Caucasian	25091	13.5	143	13.4
African-American	27671	14.9	111	10.4
Asian/Pacific	4948	2.6	32	2.9
Alaskan/American Indian	542	0.29	0	0
**GENDER**				
Female	95157	51.2	449	42
Male	90718	48.8	619	57.9
**AGE**				
0–5	35192	18.9	439	41
6–12	89948	48.3	526	49
13–18	42220	22.7	77	7.2
19–21	13749	7.4	25	2.3
22–64	4783	2.6	1	0.09
Total	185892		1068	

During the original analysis period (January–June 2016) we found ten distinct coded “concerning diagnoses” from 57 MHR patients, which could trigger referral to a clinical immunologist (Table 2). None of these 57 patients were given a PI-specific diagnosis during the 12 month follow up period (July 2016–June 2017) and while some laboratory evaluations were performed on this group [10 patients w CBC/diff & immunoglobulins (18%)] none were referred for immunological evaluation. However, 41 patients from the MHR cohort, were determined to have a PI as noted by ICD9 or ICD10 code entry in the 12 months following the original analysis and targeted intervention. (Table 3) These PI patients were detected by the Analyzer and noted to have a coded immunodeficiency along with their other claims which identified them as a new PI diagnosis not present previously in the original ICD code analysis. A high-level comparison of ICD codes between the PI and non-PI MHR cohorts is shown in Supplemental Table 3 and the full list of ICD codes for the entire MHR Cohort (PI vs. non-PI) is available as supplementary material. The 41 PI patients represent 3.8% of the longitudinally followed, focused MHR cohort of 1,068 TCH Health Plan members. In this group of PI patients, the most commonly coded immunodeficiency was “immunodeficiency Not Otherwise Specified (NOS)” (80%) and antibody deficiencies comprised 8 of the 46 coded conditions (17%).

**Table 2:** Non-PI concerning diagnostic codes found in the MHR group (*n* = 59).

**Diagnosis**	**Number (%)**
Cellulitis	18 (30)
Abscess	14 (24)
Recurrent otitis media	11 (18)
Recurrent sinusitis	5 (8)
Bacterial pneumonia	5 (8)
Osteomyelitis	2 (4)
Mastoiditis	1 (2)
Pulmonary tuberculosis	1 (2)
Lymphadenitis	1 (2)
Atypical mycobacterial infection	1 (2)

**Table 3:** PI Diagnostic codes found within the MHR group (*n* = 46).

**Diagnosis**	**Number(%)**
Immunodeficiency NOS	37(80)
Selective IgA deficiency	3 (7)
Selective IgM deficiency	3 (7)
IgG Subclass deficiency	1 (2)
Common variable immunodeficiency	1 (2)
Primary immunodeficiency associated with other Disorder	1 (2)

To better understand how our assessment and intervention may have changed behavior for at-risk individuals we assessed several metrics of healthcare utilization by MHR individuals. Within the window of follow up for the 1,068 patient MHR cohort, 950 (89%) individuals sought care in the subsequent 12 months. This included 555 individuals (52%) who visited “intervened” primary care physicians and 220 (21%) underwent laboratory evaluation over the same time frame. We could not directly correlate whether the targeted intervention letters prompted visits and laboratory assessments. Also, while referral to an immunologist was not ascertained, 35 MHR individuals (3.3%) were referred to subspecialists during the window of follow-up.

## Discussion

**Figure 2:**
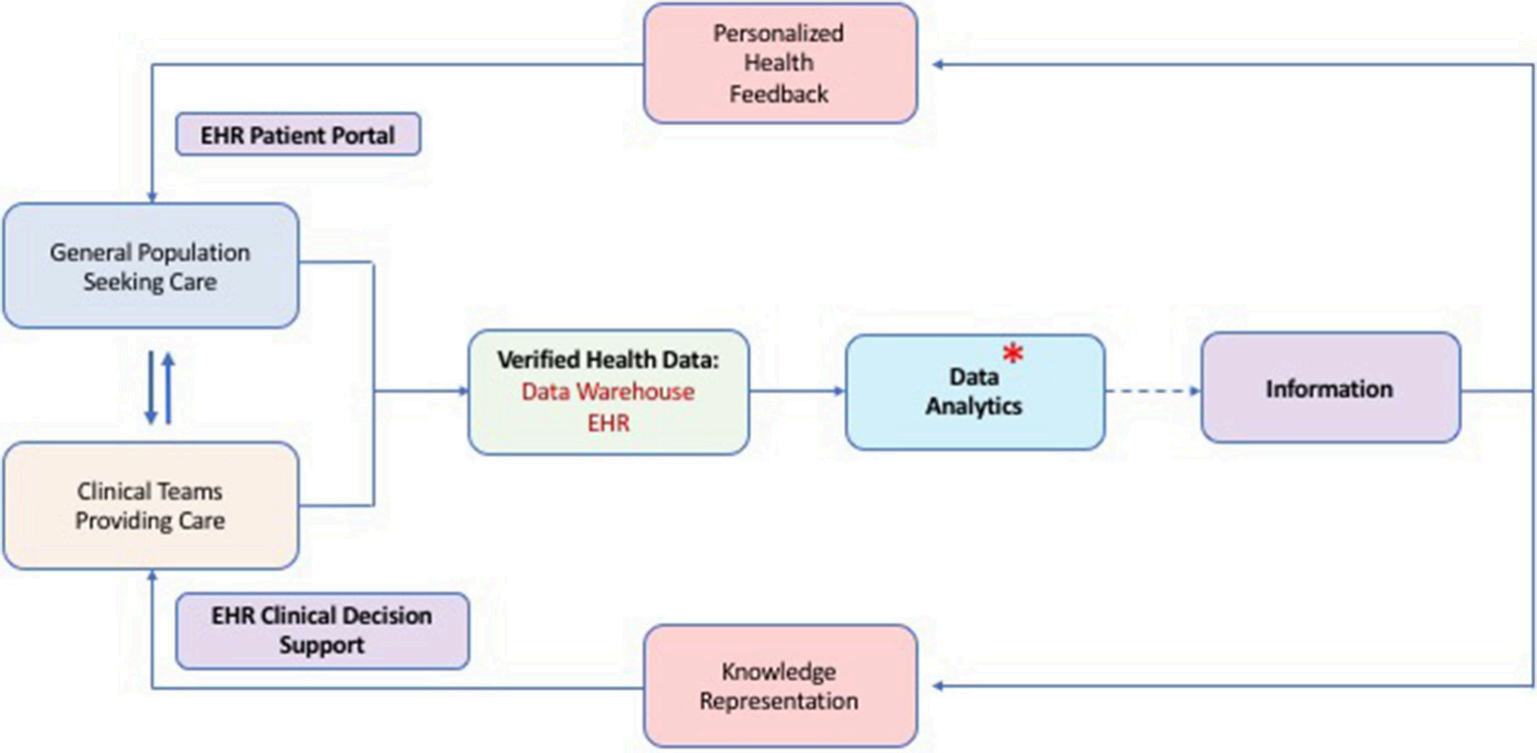
Proposed methodology for population-wide risk assessment, calculation of a risk vital sign for PI and utility of this for clinical decision support. Data flows from the clinical encounters which is subsequently verified, stored and analyzed. Analysis of quality data produces information which can be presented to patients and clinicians for optimized and shared decision making about health practices. An asterisk shows the process step where our PI risk vital sign algorithm could fit into the overall health data scheme. (EHR, Electronic Health Record).

Our present analysis provides the first large and systematic, multi-faceted study of a general population's risk for PI. It is important to note that our ICD and pharmacy claim screening approach provided risk-assessment by calculating an individual risk vital sign for PI. The algorithm cannot yet make a diagnosis of PI. However, nearly 4% of the MHR cohort was ultimately given a PI diagnosis during the 12 month follow up period and following a targeted intervention. This suggests that the algorithm is effective for identifying a higher risk group enriched for immunological dysfunction. Calling out patients with a medium-high risk “vital sign” for PI could be useful for inclusion into EHR-based CDS systems for busy clinicians. Our intentions were to assess utility of this tool in its application in a real-world health system amidst the numerous confounders of healthcare delivery in the United States. Given the efficiency and availability of informatics tools for refinement of risk, we suggest this as a viable approach for comprehensive population-wide PI risk screening and could be implemented broadly across any health system utilizing ICD coding (Figure 2). It should also be noted that this methodology is expected to facilitate healthcare provider judgement about risk of PI in their patients during a clinical encounter. The risk score would not be powered to supersede informed clinical judgement or influence insurance payer determinations.

Use of the diagnostic code Analyzer to assess risk in our general population cohort suggested that 2,188 patients (~1%) might be at risk of having a PI as shown in Figure 1. This is a greater prevalence than prior studies of PI epidemiology; however, it may represent an appropriate subsection of the general population who warrant further scrutiny of immunodeficiency risk (15, 21). Further refinement of our algorithm could sharpen the risk focus too thereby maximizing sensitivity and specificity and allowing for real-world calculation of these important measures. Use of additional informatics methodologies, including claims data analysis, could further enhance the process and reduce the focused group of interest thereby saving costs and unnecessary worry by patients and providers. A multi-pronged approach of this nature has proven effective for optimizing diagnosis in other disease states (6, 7, 22–24).

It is not surprising that most of our PI cohort is suggested to have antibody deficiency (Table 3). With the advent of T-cell Receptor Excision Circle (TREC) based newborn screening for significant T-cell deficiency, we expect that those patients will be detected early and thereby omitted from subsequent population-wide analyses such as ours (18, 25). Since the demographic of PI patients is known to be largely represented by individuals with antibody deficiency syndromes, data systems for PI risk screening should be particularly robust for assessing the likelihood of this category of host-defense impairment (15, 16, 20). It is also important to note that 80% of the PI specific codes (Table 3) were entered as “Immunodeficiency NOS” (D84.9) which could represent combined immunodeficiencies, phagocyte disorders, other well defined primary immunodeficiencies not recognized by the coder or even misdiagnosis in general.

Because our analysis was largely based on health plan claims data, we do not have complete clinical information about all of the MHR patients. However, chart review of 769 (0.4% of Main Cohort) individuals showed some level of concern by healthcare providers for immunodeficiency as noted by the codes entered and represented in Tables 2, 3. It is possible that we are missing important data from individuals in the remainder of the MHR group (1,419 patients), which were not studied at the chart level. This suggests the importance of a comprehensive and universal analytics system, which crosses health system boundaries and can provide CDS to providers independent of the health system or EHR used. This is especially important since patients may require care in different health systems for a variety of reasons. In such a scenario the portability of their complete health data should follow them to allow for continuity and a complete health data set irrespective of geographic practice location or health system accessed.

Since undiagnosed PI patients suffer excessive morbidity, mortality and healthcare spending, the 57 patients with a coded “concerning diagnosis” warrant special attention (15, 21). The costs for basic immune screening (i.e., CBC/diff, serum Immunoglobulins) in such patients will be negligible in comparison to ongoing expenses if they represent individuals with undiagnosed underlying PI. Estimates of post-diagnosis healthcare savings are noted to be $78,166 (USD) per PI patient per year even when immunoglobulin replacement therapy is taken into account (15). Additionally, mortality and costs associated with hospitalization, are higher in younger children approaching 2% and $60,000, respectively, depending upon age and type of PI (21). Based upon these reports, but not actual cost calculations in our cohort, identification of 98 PI individuals (41 with PI + 57 with concerning diagnosis) could represent an overall savings of at least $7.7 million U.S. Dollars.

## Limitations

We realize that several limitations exist in our scope and analysis. First, we don't have complete ascertainment of all identified at-risk patients. Thus, health record examination for all 2188 MHR individuals was not possible. Second, we did not have a system in place for triaging referrals to clinical immunologists to fully vet a concern about PI for high-risk individuals; thus, final determination about disease prevalence was not possible. Third, the low-risk population was not followed or studied longitudinally; therefore, it is possible that patients with disease presented after our analysis was performed. This speaks to a need for ongoing, iterative analysis using informatic tools. Fourth, our “targeted intervention” based upon individuals identified to be at risk was not controlled for. Lastly, the large number of generic “Immunodeficiency NOS” codes entered in the PI group suggests some ambiguity. It is possible that primary care physicians were uncertain about the type of PI, have historical practices of using a generic code, or used a generic code to justify billing for example. Given these limitations and knowledge gaps, we propose additional population-based studies with patients in unified health systems. Evaluation of performance of this approach for distinct classes of PI will also be important as will devising methods of artificial intelligence for refinement of analysis with time.

## Conclusions

Our study demonstrates the utility of health claims-based analysis toward risk assessment of PI in a large, diverse population. Analysis identified potentially 1% of the general population worthy of additional evaluation and upwards of 4% of a medium to high-risk population were ultimately diagnosed with PI following targeted intervention. Patients with a medium to high risk “vital sign” could be called out to busy clinicians through EHR CDS systems for further scrutiny to determine true disease risk. Given the costs and morbidity associated with undiagnosed PI patients, informed, population-wide screening is warranted and should be integrated into other health-delivery systems. This may facilitate diagnosis and improve tracking of relevant quality metrics.

## Data Availability

All relevant datasets generated for this study are included in the manuscript and/or the supplementary files.

## Ethics Statement

This study was carried out in accordance with the recommendations of Baylor College of Medicine's IRB for use of de-identified claims data and health records. The protocol was approved by the BCM IRB.

## Author Contributions

NR conceived and designed the study, drafted the initial manuscript, reviewed and revised the work. DM helped to design the study and facilitated data collection and analysis. MD performed vital data analysis. FM and VM helped design the study and provided data analysis. JQ provided important data elements and critical data analysis with methodology oversight. JO and HS helped to conceive and design the study, they also provided important intellectual content and reviewed and revised the manuscript. All authors approved the final submitted manuscript.

### Conflict of Interest Statement

Within the last 12 month JO has served as a member of the Scientific Advisory Board of ADMA Biologics, a consultant to Shire and Grifols and receives royalties from Wolters Kluwer for UpToDate. JQ, VM, and FM are employed by the Jeffrey Modell Foundation. NR is a consultant to Horizon Pharmaceuticals and receives royalties from Wolters Kluwer for UpToDate. The remaining authors declare that the research was conducted in the absence of any commercial or financial relationships that could be construed as a potential conflict of interest.
