# Supplementary material for: Calculation of a Primary Immunodeficiency “Risk Vital Sign” via Population-Wide Analysis of Claims Data to Aid in Clinical Decision Support
Source: Front Pediatr. 2019 Mar 18;7:70. doi: 10.3389/fped.2019.00070 (PMC6431644; doi:10.3389/fped.2019.00070)
Supplement: Supplementary file 1 [file Table_1.DOCX]

| ICD-9 |  | ICD-10 |  | Weight |
| --- | --- | --- | --- | --- |
| 0071 | Giardiasis | A071 | Giardiasis | 2 |
| 0338 | Whooping cough due to other specified organism | A3780 | Whooping cough due to other Bordetella species without pneumonia | 1 |
| 0339 | Whooping cough, unspecified organism | A3790 | Whooping cough, unspecified species without pneumonia | 1 |
| 0340 | Streptococcal sore throat | J020 | Streptococcal pharyngitis | 1 |
| 0340 | Streptococcal sore throat | J0300 | Acute streptococcal tonsillitis, unspecified | 1 |
| 0341 | Scarlet fever | A389 | Scarlet fever, uncomplicated | 1 |
| 035 | Erysipelas | A46 | Erysipelas | 3 |
| 0360 | Meningococcal meningitis | A390 | Meningococcal meningitis | 3 |
| 0361 | Meningococcal encephalitis | A3981 | Meningococcal encephalitis | 3 |
| 0362 | Meningococcemia | A394 | Meningococcemia, unspecified | 3 |
| 0363 | Waterhouse-Friderichsen syndrome, meningococcal | A391 | Waterhouse-Friderichsen syndrome | 3 |
| 03640 | Meningococcal carditis, unspecified | A3950 | Meningococcal carditis, unspecified | 3 |
| 03641 | Meningococcal pericarditis | A3953 | Meningococcal pericarditis | 3 |
| 03642 | Meningococcal endocarditis | A3951 | Meningococcal endocarditis | 3 |
| 03643 | Meningococcal myocarditis | A3952 | Meningococcal myocarditis | 3 |
| 03681 | Meningococcal optic neuritis | A3982 | Meningococcal retrobulbar neuritis | 3 |
| 03682 | Meningococcal arthropathy | A3983 | Meningococcal arthritis | 3 |
| 03689 | Other specified meningococcal infections | A3989 | Other meningococcal infections | 3 |
| 0369 | Meningococcal infection, unspecified | A399 | Meningococcal infection, unspecified | 3 |
| 0380 | Streptococcal septicemia | A409 | Streptococcal sepsis, unspecified | 3 |
| 03810 | Staphylococcal septicemia, unspecified | A412 | Sepsis due to unspecified staphylococcus | 3 |
| 03811 | Methicillin susceptible Staphylococcus aureus septicemia | A4101 | Sepsis due to Methicillin susceptible Staphylococcus aureus | 3 |
| 03812 | Methicillin resistant Staphylococcus aureus septicemia | A4102 | Sepsis due to Methicillin resistant Staphylococcus aureus | 3 |
| 03819 | Other Staphylococcal septicemia | A411 | Sepsis due to other specified staphylococcus | 3 |
| 0382 | Pneumococcal septicemia | A403 | Sepsis due to Streptococcus pneumoniae | 3 |
| 0383 | Septicemia due to anaerobes | A414 | Sepsis due to anaerobes | 3 |
| 03840 | Septicemia due to Gram-negative organism, unspecified | A4150 | Gram-negative sepsis, unspecified | 3 |
| 03841 | Hemophilus influenza Septicemia | A413 | Sepsis due to Hemophilus influenzae | 3 |
| 03842 | Escherichia coli septicemia | A4151 | Sepsis due to Escherichia coli [E. coli] | 3 |
| 03843 | Pseudomonas Septicemia | A4152 | Sepsis due to Pseudomonas | 3 |
| 03844 | Serratia Septicemia | A4153 | Sepsis due to Serratia | 3 |
| 03849 | Other Septicemia | A4159 | Other Gram-negative sepsis | 3 |
| 0388 | Other Specified septicemias | A4189 | Other specified sepsis | 3 |
| 0389 | Unspecified septicemia | A419 | Sepsis, unspecified organism | 3 |
| 0470 | Menningitis due to enterovirus | A870 | Enteroviral meningitis | 3 |
| 0478 | Other specified viral meningitis | A878 | Other viral meningitis | 3 |
| 0479 | Unspecified viral meningitis | A879 | Viral meningitis, unspecified | 3 |
| 0551 | Post-measles pneumonia | B052 | Measles complicated by pneumonia | 3 |
| 1120 | Candidiasis of mouth (thrush) | B370 | Candidal stomatitis | 1 |
| 1120 | Candidiasis of mouth (thrush) | B3783 | Candidal cheilitis | 1 |
| 1123 | Cutaneous candidiasis | B372 | Candidiasis of skin and nail | 1 |
| 1124 | Candidiasis of lung (candidial pneumonia) | B371 | Pulmonary candidiasis | 3 |
| 1125 | Candidiasis disseminated | B377 | Candidal sepsis | 3 |
| 11283 | Candidal meningitis | B375 | Candidal meningitis | 3 |
| 11284 | Candidal esophagitis | B3781 | Candidal esophagitis | 2 |
| 11285 | Candidal enteritis | B3782 | Candidal enteritis | 2 |
| 11289 | Candidiasis other specified site | B3789 | Other sites of candidiasis | 2 |
| 1129 | Candidiasis unspecified site | B379 | Candidiasis, unspecified | 2 |
| 1142 | Coccidioidal meningitis | B384 | Coccidioidomycosis meningitis | 3 |
| 11505 | Histoplasma capsulatum pneumonia | B392 | Pulmonary histoplasmosis capsulati, unspecified | 3 |
| 11515 | Histoplasma duboisii pneumonia | B395 | Histoplasmosis duboisii | 3 |
| 11515 | Histoplasma duboisii pneumonia | J17 | Pneumonia in diseases classified elsewhere | 3 |
| 11595 | Unspecified Histoplasmosis pneumonia | B399 | Histoplasmosis, unspecified | 3 |
| 11595 | Unspecified Histoplasmosis pneumonia | J17 | Pneumonia in diseases classified elsewhere | 3 |
| 1171 | Sporotrichosis | B420 | Pulmonary sporotrichosis | 1 |
| 1171 | Sporotrichosis | B421 | Lymphocutaneous sporotrichosis | 1 |
| 1171 | Sporotrichosis | B427 | Disseminated sporotrichosis | 1 |
| 1171 | Sporotrichosis | B429 | Sporotrichosis, unspecified | 1 |
| 1172 | Chromoblastomycosis | B439 | Chromomycosis, unspecified | 1 |
| 1173 | Aspergillosis | B449 | Aspergillosis, unspecified | 1 |
| 1174 | Mycotic mycetomas | B470 | Eumycetoma | 1 |
| 1175 | Cryptococcosis | B450 | Pulmonary cryptococcosis | 1 |
| 1175 | Cryptococcosis | B457 | Disseminated cryptococcosis | 1 |
| 1175 | Cryptococcosis | B459 | Cryptococcosis, unspecified | 1 |
| 1176 | Allescheriosis [Petriellidosis] | B482 | Allescheriasis | 1 |
| 1177 | Zygomycosis [Phycomycosis or Mucormycosis] | B469 | Zygomycosis, unspecified | 1 |
| 1178 | Infection by dematiacious fungi, [Phaehyphomycosis] | B488 | Other specified mycoses | 1 |
| 1179 | Other and unspecified mycoses | B488 | Other specified mycoses | 1 |
| 1179 | Other and unspecified mycoses | B49 | Unspecified mycosis | 1 |
| 1363 | Pneumocystosis | B59 | Pneumocystosis | 3 |
| 2830 | Autoimmune hemolytic anemia | D591 | Other autoimmune hemolytic anemias | 2 |
| 2839 | Acquired autoimmune hemolytic anemia, unspecified | D599 | Acquired hemolytic anemia, unspecified | 2 |
| 28730 | Primary Thrombocytopenic (ITP), unspecified | D6949 | Other primary thrombocytopenia | 2 |
| 28731 | Immune thrombocytopenia purpura | D693 | Immune thrombocytopenic purpura | 2 |
| 28732 | Evan's syndrome | D6941 | Evans syndrome | 2 |
| 28739 | Other primary thrombocytopenia | D693 | Immune thrombocytopenic purpura | 2 |
| 28739 | Other primary thrombocytopenia | D6949 | Other primary thrombocytopenia | 2 |
| 28800 | Neutropenia unspecified | D709 | Neutropenia, unspecified | 2 |
| 28801 | Congenital neutropenia | D700 | Congenital agranulocytosis | 2 |
| 28802 | Cyclic neutropenia | D704 | Cyclic neutropenia | 2 |
| 28804 | Neutropenia due to infection | D703 | Neutropenia due to infection | 2 |
| 28809 | Other neutropenia (immune) | D708 | Other neutropenia | 2 |
| 28850 | Leukocytopenia, unspecified | D72819 | Decreased white blood cell count, unspecified | 2 |
| 28851 | Lymphocytopenia | D72810 | Lymphocytopenia | 2 |
| 28859 | Other decreased white blood cell count | D72818 | Other decreased white blood cell count | 2 |
| 3200 | Hemophilus meningitis | G000 | Hemophilus meningitis | 3 |
| 3201 | Pneumococcal meningitis | G001 | Pneumococcal meningitis | 3 |
| 3202 | Streptococcal meningitis | G002 | Streptococcal meningitis | 3 |
| 3203 | Staphylococcal meningitis | G003 | Staphylococcal meningitis | 3 |
| 32081 | Anaerobic meningitis | G008 | Other bacterial meningitis | 3 |
| 32082 | Meningitis: Gram-negative bacteria, not elsewhere classified | G009 | Bacterial meningitis, unspecified | 3 |
| 32089 | Meningitis due to other specified bacteria | G008 | Other bacterial meningitis | 3 |
| 3209 | Meningitis due to unspecified bacterium | G009 | Bacterial meningitis, unspecified | 3 |
| 3209 | Meningitis due to unspecified bacterium | G042 | Bacterial meningoencephalitis and meningomyelitis, not elsewhere classified | 3 |
| 3220 | Nonpyogenic meningitis | G030 | Nonpyogenic meningitis | 3 |
| 3221 | Eosinophilic meningitis | G038 | Meningitis due to other specified causes | 3 |
| 3222 | Chronic meningitis | G031 | Chronic meningitis | 3 |
| 3229 | Unspecified meningitis | G039 | Meningitis, unspecified | 3 |
| 32381 | Other causes of encephalitis and encephalomyelitis | G0481 | Other encephalitis and encephalomyelitis | 3 |
| 32382 | Other causes of myelitis | G0489 | Other myelitis | 3 |
| 3239 | Unspecified causes of encephalitis, myelitis, and encephalomyelitis | G0490 | Encephalitis and encephalomyelitis, unspecified | 3 |
| 3239 | Unspecified causes of encephalitis, myelitis, and encephalomyelitis | G0491 | Myelitis, unspecified | 3 |
| 3240 | Intracarnial Abscess | G060 | Intracranial abscess and granuloma | 3 |
| 38010 | Unspecified infective otitis externa | H6000 | Abscess of external ear, unspecified ear | 1 |
| 38013 | Other acute infections of external ear | H6240 | Otitis externa in other diseases classified elsewhere, unspecified ear | 1 |
| 38015 | Chronic mycotic otitis externa | H628X1 | Other disorders of right external ear in diseases classified elsewhere | 1 |
| 38016 | Other chronic infective otitis externa | H60399 | Other infective otitis externa, unspecified ear | 1 |
| 38022 | Other acute otitis externa | H60509 | Unspecified acute noninfective otitis externa, unspecified ear | 1 |
| 38022 | Other acute otitis externa | H60519 | Acute actinic otitis externa, unspecified ear | 1 |
| 38022 | Other acute otitis externa | H60529 | Acute chemical otitis externa, unspecified ear | 1 |
| 38022 | Other acute otitis externa | H60539 | Acute contact otitis externa, unspecified ear | 1 |
| 38022 | Other acute otitis externa | H60549 | Acute eczematoid otitis externa, unspecified ear | 1 |
| 38022 | Other acute otitis externa | H60559 | Acute reactive otitis externa, unspecified ear | 1 |
| 38022 | Other acute otitis externa | H60599 | Other noninfective acute otitis externa, unspecified ear | 1 |
| 38023 | Other chronic otitis externa | H6060 | Unspecified chronic otitis externa, unspecified ear | 1 |
| 38023 | Other chronic otitis externa | H608X1 | Other otitis externa, right ear | 1 |
| 38023 | Other chronic otitis externa | H6090 | Unspecified otitis externa, unspecified ear | 1 |
| 38100 | Unspecified acute nonsuppurative otitis media | H65199 | Other acute nonsuppurative otitis media, unspecified ear | 1 |
| 38101 | Acute serous otitis media | H6500 | Acute serous otitis media, unspecified ear | 1 |
| 38102 | Acute mucoid otitis media | H65119 | Acute and subacute allergic otitis media (mucoid) (sanguinous) (serous), unspecified ear | 1 |
| 38103 | Acute sanguinous otitis media | H65119 | Acute and subacute allergic otitis media (mucoid) (sanguinous) (serous), unspecified ear | 1 |
| 38104 | Acute allergic serous otitis media | H65119 | Acute and subacute allergic otitis media (mucoid) (sanguinous) (serous), unspecified ear | 1 |
| 38105 | Acute allergic mucoid otitis media | H65119 | Acute and subacute allergic otitis media (mucoid) (sanguinous) (serous), unspecified ear | 1 |
| 38106 | Acute allergic sanguinous otitis media | H65119 | Acute and subacute allergic otitis media (mucoid) (sanguinous) (serous), unspecified ear | 1 |
| 38110 | Simple or unspecified chronic serous otitis media | H6520 | Chronic serous otitis media, unspecified ear | 1 |
| 38119 | Other chronic serous otitis media | H6520 | Chronic serous otitis media, unspecified ear | 1 |
| 38120 | Simple or unspecified chronic mucoid otitis media | H6530 | Chronic mucoid otitis media, unspecified ear | 1 |
| 38129 | Other chronic mucoid otitis media | H6530 | Chronic mucoid otitis media, unspecified ear | 1 |
| 38150 | Unspecified Eustachian salpingitis | H68009 | Unspecified Eustachian salpingitis, unspecified ear | 1 |
| 38151 | Acute Eustachian salpingitis | H68019 | Acute Eustachian salpingitis, unspecified ear | 1 |
| 38152 | Chronic Eustachian salpingitis | H68029 | Chronic Eustachian salpingitis, unspecified ear | 1 |
| 38200 | Acute suppurative otitis media without spontaneous rupture of eardrum | H66009 | Acute suppurative otitis media without spontaneous rupture of ear drum, unspecified ear | 1 |
| 3821 | Chronic tubotympanic suppurative otitis media | H6613 | Chronic tubotympanic suppurative otitis media, bilateral | 1 |
| 3822 | Chronic atticoantral suppurative otitis media | H6623 | Chronic atticoantral suppurative otitis media, bilateral | 1 |
| 3823 | Chronic suppurative otitis media | H663X9 | Other chronic suppurative otitis media, unspecified ear | 1 |
| 3824 | Suppurative otitis media | H6640 | Suppurative otitis media, unspecified, unspecified ear | 1 |
| 3829 | Chronic otitis media | H6690 | Otitis media, unspecified, unspecified ear | 1 |
| 3831 | Chronic mastoiditis | H7010 | Chronic mastoiditis, unspecified ear | 1 |
| 4610 | Acute maxillary sinusitis | J0100 | Acute maxillary sinusitis, unspecified | 1 |
| 4611 | Acute frontal sinusitis | J0110 | Acute frontal sinusitis, unspecified | 1 |
| 4612 | Acute ethmoidal sinusitis | J0120 | Acute ethmoidal sinusitis, unspecified | 1 |
| 4613 | Acute sphenoidal sinusitis | J0130 | Acute sphenoidal sinusitis, unspecified | 1 |
| 4618 | Other acute sinusitis | J0140 | Acute pansinusitis, unspecified | 1 |
| 4619 | Acute sinusitis, unspecified | J0190 | Acute sinusitis, unspecified | 1 |
| 463 | Acute tonsillitis | J0390 | Acute tonsillitis, unspecified | 1 |
| 46400 | Acute laryngitis, without mention of obstruction | J040 | Acute laryngitis | 1 |
| 46401 | Acute laryngitis, with obstruction | J050 | Acute obstructive laryngitis [croup] | 1 |
| 46410 | Acute tracheitis without mention of obstruction | J0410 | Acute tracheitis without obstruction | 1 |
| 46411 | Acute tracheitis with obstruction | J0411 | Acute tracheitis with obstruction | 1 |
| 46420 | Acute laryngotracheitis without mention of obstruction | J042 | Acute laryngotracheitis | 1 |
| 46421 | Acute laryngotracheitis with obstruction | J050 | Acute obstructive laryngitis [croup] | 1 |
| 46430 | Acute epiglottitis without mention of obstruction | J0510 | Acute epiglottitis without obstruction | 1 |
| 46431 | Acute epiglottitis with obstruction | J0511 | Acute epiglottitis with obstruction | 1 |
| 4650 | Acute laryngopharyngitis | J060 | Acute laryngopharyngitis | 1 |
| 4658 | Acute upper respiratory infections of multiple sites | J069 | Acute upper respiratory infection, unspecified | 1 |
| 4659 | Acute upper respiratory infections of unspecified site | J069 | Acute upper respiratory infection, unspecified | 1 |
| 4660 | Acute bronchitis | J209 | Acute bronchitis, unspecified | 1 |
| 46611 | Acute bronchiolitis | J210 | Acute bronchiolitis due to respiratory syncytial virus | 1 |
| 4721 | Chronic pharyngitis | J312 | Chronic pharyngitis | 1 |
| 4722 | Chronic nasopharyngitis | J311 | Chronic nasopharyngitis | 1 |
| 4730 | Chronic maxillary sinusitis | J320 | Chronic maxillary sinusitis | 1 |
| 4731 | Chronic frontal sinusitis | J321 | Chronic frontal sinusitis | 1 |
| 4732 | Chronic ethmoidal sinusitis | J322 | Chronic ethmoidal sinusitis | 1 |
| 4733 | Chronic sphenoidal sinusitis | J323 | Chronic sphenoidal sinusitis | 1 |
| 4738 | Other chronic sinusitis | J324 | Chronic pansinusitis | 1 |
| 4738 | Other chronic sinusitis | J328 | Other chronic sinusitis | 1 |
| 4739 | Unspecified chronic sinusitis | J329 | Chronic sinusitis, unspecified | 1 |
| 47400 | Chronic tonsillitis | J3501 | Chronic tonsillitis | 1 |
| 47401 | Chronic adenoiditis | J3502 | Chronic adenoiditis | 1 |
| 47402 | Chronic tonsillitis and adenoiditis | J3503 | Chronic tonsillitis and adenoiditis | 1 |
| 4800 | Pneumonia due to adenovirus | J120 | Adenoviral pneumonia | 3 |
| 4801 | Pneumonia due to respiratory syncytial virus | J121 | Respiratory syncytial virus pneumonia | 3 |
| 4802 | Pneumonia due to parainfluenza virus | J122 | Parainfluenza virus pneumonia | 3 |
| 4803 | Pneumonia due to SARS-associated coronavirus | J1281 | Pneumonia due to SARS-associated coronavirus | 3 |
| 4808 | Pneumonia due to other virus not elsewhere classified | J1289 | Other viral pneumonia | 3 |
| 4809 | Unspecified viral pneumonia | J129 | Viral pneumonia, unspecified | 3 |
| 481 | Pneumococcal pneumonia | J13 | Pneumonia due to Streptococcus pneumoniae | 3 |
| 481 | Pneumococcal pneumonia | J181 | Lobar pneumonia, unspecified organism | 3 |
| 4820 | Pneumonia due to Klebsiella Pneumoniae | J150 | Pneumonia due to Klebsiella pneumoniae | 3 |
| 4821 | Pneumonia due to Pseudomonas | J151 | Pneumonia due to Pseudomonas | 3 |
| 4822 | Pneumonia due to Haemophilus influenzae | J14 | Pneumonia due to Hemophilus influenzae | 3 |
| 48230 | Pneumonia due to Streptococcus, unspecified | J154 | Pneumonia due to other streptococci | 3 |
| 48231 | Pneumonia due to Streptococcus Group A | J154 | Pneumonia due to other streptococci | 3 |
| 48232 | Pneumonia due to Streptococcus Group B | J153 | Pneumonia due to streptococcus, group B | 3 |
| 48239 | Pneumonia due to other Streptococcus | J154 | Pneumonia due to other streptococci | 3 |
| 48240 | Pneumonia due to Staphylococcus, unspecified | J1520 | Pneumonia due to staphylococcus, unspecified | 3 |
| 48241 | Methicillin susceptible pneumonia due to Staphylococcus aureus | J15211 | Pneumonia due to Methicillin susceptible Staphylococcus aureus | 3 |
| 48242 | Methicillin resistant pneumonia due to Staphylococcus aureus | J15212 | Pneumonia due to Methicillin resistant Staphylococcus aureus | 3 |
| 48249 | Pneumonia due to other staphylococcus pneumonia | J1529 | Pneumonia due to other staphylococcus | 3 |
| 48281 | Pneumonia due to anaerobes | J158 | Pneumonia due to other specified bacteria | 3 |
| 48282 | Pneumonia due to E. coli | J155 | Pneumonia due to Escherichia coli | 3 |
| 48283 | Pneumonia due to other gram-negative bacteria | J156 | Pneumonia due to other aerobic Gram-negative bacteria | 3 |
| 48284 | Pneumonia due to Legionnaires' disease | A481 | Legionnaires' disease | 3 |
| 48289 | Pneumonia due to other specified bacteria | J158 | Pneumonia due to other specified bacteria | 3 |
| 4829 | Bacterial pneumonia, unspecified | J159 | Unspecified bacterial pneumonia | 3 |
| 4830 | Pneumonia due to Mycoplasma pneumoniae | J157 | Pneumonia due to Mycoplasma pneumoniae | 3 |
| 4831 | Pneumonia due to Chlamydia | J160 | Chlamydial pneumonia | 3 |
| 4838 | Pneumonia due to other specified organism | J168 | Pneumonia due to other specified infectious organisms | 3 |
| 4841 | Pneumonia in cytomegalic inclusion disease | B250 | Cytomegaloviral pneumonitis | 3 |
| 4843 | Pneumonia in whooping cough | A3791 | Whooping cough, unspecified species with pneumonia | 3 |
| 4846 | Pneumonia in aspergillosis | B440 | Invasive pulmonary aspergillosis | 3 |
| 4847 | Pneumonia in other systemic mycoses | J17 | Pneumonia in diseases classified elsewhere | 3 |
| 4848 | Pneumonia in other infectious diseases classified elsewhere | J17 | Pneumonia in diseases classified elsewhere | 3 |
| 485 | Bronchopneumonia organism unspecified | J180 | Bronchopneumonia, unspecified organism | 3 |
| 486 | Pneumonia, organism not otherwise specified | J189 | Pneumonia, unspecified organism | 3 |
| 4870 | Influenza with pneumonia | J1100 | Influenza due to unidentified influenza virus with unspecified type of pneumonia | 3 |
| 4870 | Influenza with pneumonia | J129 | Viral pneumonia, unspecified | 3 |
| 4910 | Chronic bronchitis. Excludes:. chronic obstructive asthma | J410 | Simple chronic bronchitis | 1 |
| 4911 | Mucopurulent chronic bronchitis | J411 | Mucopurulent chronic bronchitis | 1 |
| 4919 | Bronchitis - Chronic & Unspecified | J42 | Unspecified chronic bronchitis | 1 |
| 4940 | Bronchiectasis without acute exacerbation | J479 | Bronchiectasis, uncomplicated | 2 |
| 4941 | Bronchiectasis with acute exacerbation | J471 | Bronchiectasis with (acute) exacerbation | 2 |
| 5100 | Empyema with fistula | J860 | Pyothorax with fistula | 3 |
| 5109 | Empyema without mention of fistula | J869 | Pyothorax without fistula | 3 |
| 5130 | Abscess of lung | J850 | Gangrene and necrosis of lung | 3 |
| 5130 | Abscess of lung | J851 | Abscess of lung with pneumonia | 3 |
| 5130 | Abscess of lung | J852 | Abscess of lung without pneumonia | 3 |
| 5131 | Abscess of lung and mediastinum | J853 | Abscess of mediastinum | 3 |
| 5168 | Bronchiolitis Obliterans Organized Pneumonia (BOOP) A | J8409 | Other alveolar and parieto-alveolar conditions | 3 |
| 5273 | Abscess of salivary gland | K113 | Abscess of salivary gland | 3 |
| 5283 | Cellulitis and abscess of oral soft tissues | K122 | Cellulitis and abscess of mouth | 3 |
| 5401 | Acute appendicitis with peritoneal abscess | K353 | Acute appendicitis with localized peritonitis | 3 |
| 566 | Anal and rectal abscess | K610 | Anal abscess | 3 |
| 566 | Anal and rectal abscess | K611 | Rectal abscess | 3 |
| 566 | Anal and rectal abscess | K613 | Ischiorectal abscess | 3 |
| 56722 | Peritoneal abscess | K651 | Peritoneal abscess | 3 |
| 5720 | Abscess of liver | K750 | Abscess of liver | 3 |
| 5799 | Unspecified intestinal malabsorption | K909 | Intestinal malabsorption, unspecified | 1 |
| 5902 | Renal and perinephric abscess | N151 | Renal and perinephric abscess | 3 |
| 5970 | Urethral abscess | N340 | Urethral abscess | 3 |
| 6040 | Orchitis, epididymitis, and epididymo-orchitis, with abscess | N454 | Abscess of epididymis or testis | 3 |
| 6144 | Chronic or unspecified parametritis and pelvic cellulitis | N731 | Chronic parametritis and pelvic cellulitis | 2 |
| 6144 | Chronic or unspecified parametritis and pelvic cellulitis | N732 | Unspecified parametritis and pelvic cellulitis | 2 |
| 6163 | Abscess of Bartholin's gland | N751 | Abscess of Bartholin's gland | 3 |
| 6164 | Other abscess of vulva | N764 | Abscess of vulva | 3 |
| 6820 | Cellulitis and abscess of face | K122 | Cellulitis and abscess of mouth | 2 |
| 6820 | Cellulitis and abscess of face | L03211 | Cellulitis of face | 2 |
| 6820 | Cellulitis and abscess of face | L03212 | Acute lymphangitis of face | 2 |
| 6821 | Cellulitis and abscess of neck | L03221 | Cellulitis of neck | 2 |
| 6821 | Cellulitis and abscess of neck | L03222 | Acute lymphangitis of neck | 2 |
| 6822 | Cellulitis and abscess of trunk | L03319 | Cellulitis of trunk, unspecified | 2 |
| 6822 | Cellulitis and abscess of trunk | L03329 | Acute lymphangitis of trunk, unspecified | 2 |
| 6823 | Cellulitis and abscess of upper arm and forearm | L03119 | Cellulitis of unspecified part of limb | 2 |
| 6823 | Cellulitis and abscess of upper arm and forearm | L03129 | Acute lymphangitis of unspecified part of limb | 2 |
| 6824 | Cellulitis and abscess of hand, except fingers and thumb | L03119 | Cellulitis of unspecified part of limb | 2 |
| 6824 | Cellulitis and abscess of hand, except fingers and thumb | L03129 | Acute lymphangitis of unspecified part of limb | 2 |
| 6825 | Cellulitis and abscess of buttock | L03317 | Cellulitis of buttock | 2 |
| 6826 | Cellulitis and abscess of leg, except foot | L03119 | Cellulitis of unspecified part of limb | 2 |
| 6826 | Cellulitis and abscess of leg, except foot | L03129 | Acute lymphangitis of unspecified part of limb | 2 |
| 6827 | Cellulitis and abscess of foot, except toes | L03119 | Cellulitis of unspecified part of limb | 2 |
| 6827 | Cellulitis and abscess of foot, except toes | L03129 | Acute lymphangitis of unspecified part of limb | 2 |
| 6828 | Cellulitis and abscess of other specified sites | L03811 | Cellulitis of head [any part, except face] | 2 |
| 6828 | Cellulitis and abscess of other specified sites | L03818 | Cellulitis of other sites | 2 |
| 6828 | Cellulitis and abscess of other specified sites | L03891 | Acute lymphangitis of head [any part, except face] | 2 |
| 6828 | Cellulitis and abscess of other specified sites | L03898 | Acute lymphangitis of other sites | 2 |
| 6829 | Cellulitis unspecified site | L0390 | Cellulitis, unspecified | 2 |
| 6829 | Cellulitis unspecified site | L0391 | Acute lymphangitis, unspecified | 2 |
| 683 | Acute lymphadenitis | L049 | Acute lymphadenitis, unspecified | 2 |
| 6850 | Pilonidal cyst with abscess | L0501 | Pilonidal cyst with abscess | 3 |
| 6850 | Pilonidal cyst with abscess | L0502 | Pilonidal sinus with abscess | 3 |
| 6918 | Atopic dermatitis | L200 | Besnier's prurigo | 1 |
| 6918 | Atopic dermatitis | L2081 | Atopic neurodermatitis | 1 |
| 6918 | Atopic dermatitis | L2082 | Flexural eczema | 1 |
| 6918 | Atopic dermatitis | L2084 | Intrinsic (allergic) eczema | 1 |
| 6918 | Atopic dermatitis | L2089 | Other atopic dermatitis | 1 |
| 73000 | Acute osteomyelitis, site unspecified | M8610 | Other acute osteomyelitis, unspecified site | 3 |
| 73000 | Acute osteomyelitis, site unspecified | M8620 | Subacute osteomyelitis, unspecified site | 3 |
| 73001 | Acute osteomyelitis, shoulder region | M86119 | Other acute osteomyelitis, unspecified shoulder | 3 |
| 73001 | Acute osteomyelitis, shoulder region | M86219 | Subacute osteomyelitis, unspecified shoulder | 3 |
| 73002 | Acute osteomyelitis, upper arm | M86129 | Other acute osteomyelitis, unspecified humerus | 3 |
| 73002 | Acute osteomyelitis, upper arm | M86229 | Subacute osteomyelitis, unspecified humerus | 3 |
| 73003 | Acute osteomyelitis, forearm | M86139 | Other acute osteomyelitis, unspecified radius and ulna | 3 |
| 73003 | Acute osteomyelitis, forearm | M86239 | Subacute osteomyelitis, unspecified radius and ulna | 3 |
| 73004 | Acute osteomyelitis, hand | M86149 | Other acute osteomyelitis, unspecified hand | 3 |
| 73004 | Acute osteomyelitis, hand | M86249 | Subacute osteomyelitis, unspecified hand | 3 |
| 73005 | Acute osteomyelitis, pelvic region and thigh | M86159 | Other acute osteomyelitis, unspecified femur | 3 |
| 73005 | Acute osteomyelitis, pelvic region and thigh | M86259 | Subacute osteomyelitis, unspecified femur | 3 |
| 73006 | Acute osteomyelitis, lower leg | M86169 | Other acute osteomyelitis, unspecified tibia and fibula | 3 |
| 73006 | Acute osteomyelitis, lower leg | M86269 | Subacute osteomyelitis, unspecified tibia and fibula | 3 |
| 73007 | Acute osteomyelitis, ankle and foot | M86179 | Other acute osteomyelitis, unspecified ankle and foot | 3 |
| 73007 | Acute osteomyelitis, ankle and foot | M86279 | Subacute osteomyelitis, unspecified ankle and foot | 3 |
| 73008 | Acute osteomyelitis, other specified sites | M8618 | Other acute osteomyelitis, other site | 3 |
| 73008 | Acute osteomyelitis, other specified sites | M8628 | Subacute osteomyelitis, other site | 3 |
| 73009 | Acute osteomyelitis, multiple sites | M8619 | Other acute osteomyelitis, multiple sites | 3 |
| 73009 | Acute osteomyelitis, multiple sites | M8629 | Subacute osteomyelitis, multiple sites | 3 |
| 73010 | Chronic osteomyelitis, site unspecified | M8660 | Other chronic osteomyelitis, unspecified site | 3 |
| 73011 | Chronic osteomyelitis, shoulder region | M86619 | Other chronic osteomyelitis, unspecified shoulder | 3 |
| 73012 | Chronic osteomyelitis, upper arm | M86629 | Other chronic osteomyelitis, unspecified humerus | 3 |
| 73013 | Chronic osteomyelitis, forearm | M86639 | Other chronic osteomyelitis, unspecified radius and ulna | 3 |
| 73014 | Chronic osteomyelitis, hand | M86642 | Other chronic osteomyelitis, left hand | 3 |
| 73015 | Chronic osteomyelitis, pelvic region and thigh | M86659 | Other chronic osteomyelitis, unspecified thigh | 3 |
| 73016 | Chronic osteomyelitis, lower leg | M86669 | Other chronic osteomyelitis, unspecified tibia and fibula | 3 |
| 73017 | Chronic osteomyelitis, ankle and foot | M86679 | Other chronic osteomyelitis, unspecified ankle and foot | 3 |
| 73018 | Chronic osteomyelitis, other specified sites | M8668 | Other chronic osteomyelitis, other site | 3 |
| 73019 | Chronic osteomyelitis, multiple sites | M8669 | Other chronic osteomyelitis, multiple sites | 3 |
| 73020 | Unspecified osteomyelitis, site unspecified | M869 | Osteomyelitis, unspecified | 3 |
| 73021 | Unspecified osteomyelitis, shoulder region | M869 | Osteomyelitis, unspecified | 3 |
| 73022 | Unspecified osteomyelitis, upper arm | M869 | Osteomyelitis, unspecified | 3 |
| 73023 | Unspecified osteomyelitis, forearm | M869 | Osteomyelitis, unspecified | 3 |
| 73024 | Unspecified osteomyelitis, hand | M869 | Osteomyelitis, unspecified | 3 |
| 73025 | Unspecified osteomyelitis, pelvic region and thigh | M869 | Osteomyelitis, unspecified | 3 |
| 73026 | Unspecified osteomyelitis, lower leg | M869 | Osteomyelitis, unspecified | 3 |
| 73027 | Unspecified osteomyelitis, ankle and foot | M869 | Osteomyelitis, unspecified | 3 |
| 73028 | Unspecified osteomyelitis, other specified sites | M4620 | Osteomyelitis of vertebra, site unspecified | 3 |
| 73029 | Unspecified osteomyelitis, multiple sites | M869 | Osteomyelitis, unspecified | 3 |
| 78321 | Abnormal loss of weight | R634 | Abnormal weight loss | 1 |
| 78340 | Lack normal physiologic development | R6250 | Unspecified lack of expected normal physiological development in childhood | 1 |
| 78341 | Failure to thrive (failure to gain weight) | R6251 | Failure to thrive (child) | 1 |
| 7856 | Lymphadenopathy | R599 | Enlarged lymph nodes, unspecified | 1 |
| 78791 | Diarrhea | K522 | Allergic and dietetic gastroenteritis and colitis | 1 |
| 78791 | Diarrhea | K5289 | Other specified noninfective gastroenteritis and colitis | 1 |
| 78791 | Diarrhea | R197 | Diarrhea, unspecified | 1 |
| 7892 | Splenomegaly | R161 | Splenomegaly, not elsewhere classified | 2 |
| V0251 | Carrier or suspected carrier Group B streptococcus | Z22330 | Carrier of Group B streptococcus | 1 |
| V0252 | Carrier or suspected carrier other streptococcus | Z22338 | Carrier of other streptococcus | 1 |
| V0253 | Carrier or suspected carrier Methicillin susceptible Staphylococcus aureus | Z22321 | Carrier or suspected carrier of Methicillin susceptible Staphylococcus aureus | 1 |
| V0254 | Carrier or suspected carrier Methicillin resistant Staphylococcus aureus | Z22322 | Carrier or suspected carrier of Methicillin resistant Staphylococcus aureus | 2 |
| V0259 | Carrier or suspected carrier Other specified bacterial diseases | Z2231 | Carrier of bacterial disease due to meningococci | 1 |
| V0259 | Carrier or suspected carrier Other specified bacterial diseases | Z2239 | Carrier of other specified bacterial diseases | 1 |
| V090 | Infection with microorganisms resistant to penicillins | Z1611 | Resistance to penicillins | 2 |
| V091 | Infection with microorganisms resistant to cephalosporins and other B-lactam antibiotics | Z1610 | Resistance to unspecified beta lactam antibiotics | 2 |
| V091 | Infection with microorganisms resistant to cephalosporins and other B-lactam antibiotics | Z1612 | Extended spectrum beta lactamase (ESBL) resistance | 2 |
| V091 | Infection with microorganisms resistant to cephalosporins and other B-lactam antibiotics | Z1619 | Resistance to other specified beta lactam antibiotics | 2 |
| V092 | Infection with microorganisms resistant to macrolides | Z1629 | Resistance to other single specified antibiotic | 2 |
| V093 | Infection with microorganisms resistant to tetracyclines | Z1629 | Resistance to other single specified antibiotic | 2 |
| V094 | Infection with microorganisms resistant to aminoglycosides | Z1629 | Resistance to other single specified antibiotic | 2 |
| V0950 | Infection with microorganisms resistant to quinolones and fluoroquinolones, without mention of resistance to multiple quinolones and fluoroquinolones | Z1623 | Resistance to quinolones and fluoroquinolones | 2 |
| V0951 | Infection with microorganisms resistant to quinolones and fluoroquinolones, with resistance to multiple quinolones and fluoroquinolones | Z1623 | Resistance to quinolones and fluoroquinolones | 2 |
| V096 | Infection with microorganisms resistant to sulfonamides | Z1629 | Resistance to other single specified antibiotic | 2 |
| V0970 | Infection with microorganisms resistant to other specified antimycobacterial agents, without mention of resistance to antimycobacterial agents | Z16341 | Resistance to single antimycobacterial drug | 2 |
| V0971 | Infection with microorganisms resistant to other specified antimycobacterial agents, with resistance to antimycobacterial agents | Z16342 | Resistance to multiple antimycobacterial drugs | 2 |
| V0980 | Infection with microorganisms resistant to other specified drugs | Z1620 | Resistance to unspecified antibiotic | 2 |
| V0980 | Infection with microorganisms resistant to other specified drugs | Z1621 | Resistance to vancomycin | 2 |
| V0980 | Infection with microorganisms resistant to other specified drugs | Z1622 | Resistance to vancomycin related antibiotics | 2 |
| V0980 | Infection with microorganisms resistant to other specified drugs | Z1631 | Resistance to antiparasitic drug(s) | 2 |
| V0980 | Infection with microorganisms resistant to other specified drugs | Z1632 | Resistance to antifungal drug(s) | 2 |
| V0980 | Infection with microorganisms resistant to other specified drugs | Z1633 | Resistance to antiviral drug(s) | 2 |
| V0980 | Infection with microorganisms resistant to other specified drugs | Z1639 | Resistance to other specified antimicrobial drug | 2 |
| V0981 | Infection with microorganisms resistant to other specified drugs, without mention of resistance to multiple drugs | Z1635 | Resistance to multiple antimicrobial drugs | 2 |
| V0990 | Infection with microorganisms resistant to other specified drugs, without mention of resistance to multiple drugs | Z1630 | Resistance to unspecified antimicrobial drugs | 2 |
| V0991 | Infection with microorganisms resistant to other specified drugs, with resistance to multiple drugs | Z1624 | Resistance to multiple antibiotics | 2 |
| V851 | Body Mass Index, pediatric, less than 5th percentile for age C | Z6820 | Body mass index (BMI) 20.0-20.9, adult | 1 |
| V851 | Body Mass Index, pediatric, less than 5th percentile for age C | Z6821 | Body mass index (BMI) 21.0-21.9, adult | 1 |
| V851 | Body Mass Index, pediatric, less than 5th percentile for age C | Z6822 | Body mass index (BMI) 22.0-22.9, adult | 1 |
| V851 | Body Mass Index, pediatric, less than 5th percentile for age C | Z6823 | Body mass index (BMI) 23.0-23.9, adult | 1 |
| V851 | Body Mass Index, pediatric, less than 5th percentile for age C | Z6824 | Body mass index (BMI) 24.0-24.9, adult | 1 |
