# Supplementary material for: Calculation of a Primary Immunodeficiency “Risk Vital Sign” via Population-Wide Analysis of Claims Data to Aid in Clinical Decision Support
Source: Front Pediatr. 2019 Mar 18;7:70. doi: 10.3389/fped.2019.00070 (PMC6431644; doi:10.3389/fped.2019.00070)
Supplement: Supplementary file 2 [file Table_2.DOCX]

| **Supplemental Table 2: Risk Score Values for MHR Cohort** | | | |
| --- | --- | --- | --- |
| **Patient Count** | **PI Risk Category** | **Total Risk Score** | **Number of PI Warning Signs** |
| 1 | HIGH | 39 | 1 |
| 2 | HIGH | 27 | 1 |
| 3 | HIGH | 27 | 1 |
| 4 | HIGH | 26 | 1 |
| 5 | HIGH | 25 | 1 |
| 6 | HIGH | 23 | 3 |
| 7 | HIGH | 22 | 2 |
| 8 | HIGH | 21 | 2 |
| 9 | HIGH | 21 | 2 |
| 10 | HIGH | 20 | 2 |
| 11 | HIGH | 20 | 2 |
| 12 | HIGH | 20 | 3 |
| 13 | HIGH | 20 | 1 |
| 14 | HIGH | 20 | 3 |
| 15 | HIGH | 19 | 2 |
| 16 | HIGH | 19 | 1 |
| 17 | HIGH | 19 | 2 |
| 18 | HIGH | 19 | 2 |
| 19 | HIGH | 19 | 2 |
| 20 | HIGH | 19 | 1 |
| 21 | HIGH | 18 | 2 |
| 22 | HIGH | 18 | 1 |
| 23 | HIGH | 18 | 2 |
| 24 | HIGH | 18 | 1 |
| 25 | HIGH | 18 | 2 |
| 26 | HIGH | 18 | 3 |
| 27 | HIGH | 18 | 2 |
| 28 | HIGH | 18 | 2 |
| 29 | HIGH | 18 | 1 |
| 30 | HIGH | 17 | 3 |
| 31 | HIGH | 17 | 2 |
| 32 | HIGH | 17 | 1 |
| 33 | HIGH | 17 | 2 |
| 34 | HIGH | 17 | 2 |
| 35 | HIGH | 17 | 2 |
| 36 | HIGH | 16 | 1 |
| 37 | HIGH | 16 | 2 |
| 38 | HIGH | 16 | 1 |
| 39 | HIGH | 16 | 1 |
| 40 | HIGH | 16 | 1 |
| 41 | HIGH | 16 | 2 |
| 42 | HIGH | 16 | 2 |
| 43 | HIGH | 16 | 1 |
| 44 | HIGH | 16 | 1 |
| 45 | HIGH | 16 | 2 |
| 46 | HIGH | 16 | 2 |
| 47 | HIGH | 16 | 3 |
| 48 | HIGH | 16 | 1 |
| 49 | HIGH | 16 | 1 |
| 50 | HIGH | 16 | 2 |
| 51 | HIGH | 16 | 2 |
| 52 | HIGH | 16 | 2 |
| 53 | HIGH | 16 | 1 |
| 54 | HIGH | 16 | 1 |
| 55 | HIGH | 15 | 2 |
| 56 | HIGH | 15 | 1 |
| 57 | HIGH | 15 | 1 |
| 58 | HIGH | 15 | 2 |
| 59 | HIGH | 15 | 2 |
| 60 | HIGH | 15 | 1 |
| 61 | HIGH | 15 | 1 |
| 62 | HIGH | 15 | 2 |
| 63 | HIGH | 15 | 1 |
| 64 | HIGH | 15 | 1 |
| 65 | HIGH | 15 | 3 |
| 66 | HIGH | 15 | 2 |
| 67 | HIGH | 15 | 1 |
| 68 | HIGH | 15 | 1 |
| 69 | HIGH | 15 | 1 |
| 70 | HIGH | 15 | 1 |
| 71 | HIGH | 15 | 1 |
| 72 | HIGH | 15 | 2 |
| 73 | HIGH | 15 | 3 |
| 74 | HIGH | 15 | 1 |
| 75 | HIGH | 15 | 1 |
| 76 | HIGH | 15 | 1 |
| 77 | HIGH | 15 | 3 |
| 78 | HIGH | 15 | 1 |
| 79 | HIGH | 15 | 2 |
| 80 | HIGH | 14 | 2 |
| 81 | HIGH | 14 | 2 |
| 82 | HIGH | 14 | 1 |
| 83 | HIGH | 14 | 2 |
| 84 | HIGH | 14 | 2 |
| 85 | HIGH | 14 | 1 |
| 86 | HIGH | 14 | 2 |
| 87 | HIGH | 14 | 1 |
| 88 | HIGH | 14 | 2 |
| 89 | HIGH | 14 | 2 |
| 90 | HIGH | 14 | 1 |
| 91 | HIGH | 14 | 1 |
| 92 | HIGH | 14 | 1 |
| 93 | HIGH | 14 | 1 |
| 94 | HIGH | 14 | 1 |
| 95 | HIGH | 14 | 1 |
| 96 | HIGH | 14 | 1 |
| 97 | HIGH | 14 | 1 |
| 98 | HIGH | 14 | 2 |
| 99 | HIGH | 14 | 1 |
| 100 | HIGH | 14 | 2 |
| 101 | HIGH | 14 | 1 |
| 102 | HIGH | 14 | 1 |
| 103 | HIGH | 14 | 2 |
| 104 | HIGH | 14 | 2 |
| 105 | HIGH | 14 | 1 |
| 106 | HIGH | 13 | 1 |
| 107 | HIGH | 13 | 2 |
| 108 | HIGH | 13 | 2 |
| 109 | HIGH | 13 | 1 |
| 110 | HIGH | 13 | 2 |
| 111 | HIGH | 13 | 1 |
| 112 | HIGH | 13 | 1 |
| 113 | HIGH | 13 | 2 |
| 114 | HIGH | 13 | 1 |
| 115 | HIGH | 13 | 1 |
| 116 | HIGH | 13 | 2 |
| 117 | HIGH | 13 | 2 |
| 118 | HIGH | 13 | 1 |
| 119 | HIGH | 13 | 1 |
| 120 | HIGH | 13 | 2 |
| 121 | HIGH | 13 | 1 |
| 122 | HIGH | 13 | 1 |
| 123 | HIGH | 13 | 1 |
| 124 | HIGH | 13 | 1 |
| 125 | HIGH | 13 | 2 |
| 126 | HIGH | 13 | 1 |
| 127 | HIGH | 13 | 1 |
| 128 | HIGH | 13 | 1 |
| 129 | HIGH | 13 | 2 |
| 130 | HIGH | 13 | 1 |
| 131 | HIGH | 13 | 1 |
| 132 | HIGH | 13 | 1 |
| 133 | HIGH | 13 | 1 |
| 134 | HIGH | 13 | 1 |
| 135 | HIGH | 13 | 1 |
| 136 | HIGH | 13 | 1 |
| 137 | HIGH | 13 | 1 |
| 138 | HIGH | 13 | 1 |
| 139 | HIGH | 13 | 1 |
| 140 | HIGH | 13 | 1 |
| 141 | HIGH | 13 | 2 |
| 142 | HIGH | 13 | 2 |
| 143 | HIGH | 13 | 3 |
| 144 | HIGH | 13 | 2 |
| 145 | HIGH | 13 | 1 |
| 146 | HIGH | 13 | 2 |
| 147 | HIGH | 13 | 2 |
| 148 | HIGH | 13 | 1 |
| 149 | HIGH | 13 | 1 |
| 150 | HIGH | 13 | 1 |
| 151 | HIGH | 13 | 1 |
| 152 | HIGH | 13 | 1 |
| 153 | HIGH | 13 | 1 |
| 154 | HIGH | 13 | 1 |
| 155 | HIGH | 13 | 1 |
| 156 | HIGH | 13 | 1 |
| 157 | HIGH | 13 | 2 |
| 158 | HIGH | 13 | 2 |
| 159 | HIGH | 13 | 2 |
| 160 | HIGH | 13 | 1 |
| 161 | HIGH | 13 | 1 |
| 162 | HIGH | 12 | 1 |
| 163 | HIGH | 12 | 1 |
| 164 | HIGH | 12 | 1 |
| 165 | HIGH | 12 | 1 |
| 166 | HIGH | 12 | 1 |
| 167 | HIGH | 12 | 1 |
| 168 | HIGH | 12 | 1 |
| 169 | HIGH | 12 | 1 |
| 170 | HIGH | 12 | 1 |
| 171 | HIGH | 12 | 2 |
| 172 | HIGH | 12 | 1 |
| 173 | HIGH | 12 | 1 |
| 174 | HIGH | 12 | 1 |
| 175 | HIGH | 12 | 1 |
| 176 | HIGH | 12 | 1 |
| 177 | HIGH | 12 | 1 |
| 178 | HIGH | 12 | 1 |
| 179 | HIGH | 12 | 1 |
| 180 | HIGH | 12 | 1 |
| 181 | HIGH | 12 | 1 |
| 182 | HIGH | 12 | 1 |
| 183 | HIGH | 12 | 1 |
| 184 | HIGH | 12 | 1 |
| 185 | HIGH | 12 | 1 |
| 186 | HIGH | 12 | 2 |
| 187 | HIGH | 12 | 1 |
| 188 | HIGH | 12 | 1 |
| 189 | HIGH | 12 | 1 |
| 190 | HIGH | 12 | 1 |
| 191 | HIGH | 12 | 1 |
| 192 | HIGH | 12 | 1 |
| 193 | HIGH | 12 | 1 |
| 194 | HIGH | 12 | 1 |
| 195 | HIGH | 12 | 1 |
| 196 | HIGH | 12 | 1 |
| 197 | HIGH | 12 | 2 |
| 198 | HIGH | 12 | 1 |
| 199 | HIGH | 12 | 2 |
| 200 | HIGH | 12 | 1 |
| 201 | HIGH | 12 | 2 |
| 202 | HIGH | 12 | 1 |
| 203 | HIGH | 12 | 1 |
| 204 | HIGH | 12 | 1 |
| 205 | HIGH | 12 | 2 |
| 206 | HIGH | 12 | 2 |
| 207 | HIGH | 12 | 1 |
| 208 | HIGH | 12 | 3 |
| 209 | HIGH | 12 | 2 |
| 210 | HIGH | 12 | 1 |
| 211 | HIGH | 12 | 1 |
| 212 | HIGH | 12 | 3 |
| 213 | HIGH | 12 | 1 |
| 214 | HIGH | 12 | 1 |
| 215 | HIGH | 12 | 1 |
| 216 | HIGH | 12 | 2 |
| 217 | HIGH | 12 | 1 |
| 218 | HIGH | 12 | 1 |
| 219 | HIGH | 12 | 1 |
| 220 | HIGH | 12 | 2 |
| 221 | HIGH | 12 | 2 |
| 222 | HIGH | 12 | 1 |
| 223 | HIGH | 12 | 2 |
| 224 | HIGH | 12 | 1 |
| 225 | HIGH | 12 | 1 |
| 226 | HIGH | 12 | 1 |
| 227 | HIGH | 12 | 1 |
| 228 | HIGH | 12 | 1 |
| 229 | HIGH | 12 | 1 |
| 230 | HIGH | 12 | 2 |
| 231 | HIGH | 12 | 2 |
| 232 | HIGH | 12 | 1 |
| 233 | HIGH | 12 | 1 |
| 234 | HIGH | 12 | 1 |
| 235 | HIGH | 12 | 2 |
| 236 | HIGH | 12 | 1 |
| 237 | HIGH | 12 | 1 |
| 238 | HIGH | 12 | 1 |
| 239 | HIGH | 12 | 1 |
| 240 | HIGH | 12 | 1 |
| 241 | HIGH | 12 | 1 |
| 242 | HIGH | 12 | 1 |
| 243 | HIGH | 12 | 1 |
| 244 | HIGH | 12 | 1 |
| 245 | HIGH | 12 | 1 |
| 246 | HIGH | 12 | 1 |
| 247 | HIGH | 12 | 1 |
| 248 | HIGH | 12 | 2 |
| 249 | HIGH | 12 | 1 |
| 250 | HIGH | 11 | 1 |
| 251 | HIGH | 11 | 1 |
| 252 | HIGH | 11 | 1 |
| 253 | HIGH | 11 | 2 |
| 254 | HIGH | 11 | 1 |
| 255 | HIGH | 11 | 1 |
| 256 | HIGH | 11 | 1 |
| 257 | HIGH | 11 | 1 |
| 258 | HIGH | 11 | 1 |
| 259 | HIGH | 11 | 1 |
| 260 | HIGH | 11 | 1 |
| 261 | HIGH | 11 | 1 |
| 262 | HIGH | 11 | 1 |
| 263 | HIGH | 11 | 1 |
| 264 | HIGH | 11 | 1 |
| 265 | HIGH | 11 | 1 |
| 266 | HIGH | 11 | 1 |
| 267 | HIGH | 11 | 1 |
| 268 | HIGH | 11 | 1 |
| 269 | HIGH | 11 | 1 |
| 270 | HIGH | 11 | 1 |
| 271 | HIGH | 11 | 1 |
| 272 | HIGH | 11 | 1 |
| 273 | HIGH | 11 | 1 |
| 274 | HIGH | 11 | 1 |
| 275 | HIGH | 11 | 2 |
| 276 | HIGH | 11 | 1 |
| 277 | HIGH | 11 | 1 |
| 278 | HIGH | 11 | 1 |
| 279 | HIGH | 11 | 2 |
| 280 | HIGH | 11 | 1 |
| 281 | HIGH | 11 | 1 |
| 282 | HIGH | 11 | 1 |
| 283 | HIGH | 11 | 1 |
| 284 | HIGH | 11 | 1 |
| 285 | HIGH | 11 | 1 |
| 286 | HIGH | 11 | 3 |
| 287 | HIGH | 11 | 1 |
| 288 | HIGH | 11 | 1 |
| 289 | HIGH | 11 | 1 |
| 290 | HIGH | 11 | 1 |
| 291 | HIGH | 11 | 1 |
| 292 | HIGH | 11 | 1 |
| 293 | HIGH | 11 | 1 |
| 294 | HIGH | 11 | 2 |
| 295 | HIGH | 11 | 1 |
| 296 | HIGH | 11 | 1 |
| 297 | HIGH | 11 | 1 |
| 298 | HIGH | 11 | 1 |
| 299 | HIGH | 11 | 1 |
| 300 | HIGH | 11 | 2 |
| 301 | HIGH | 11 | 1 |
| 302 | HIGH | 11 | 1 |
| 303 | HIGH | 11 | 1 |
| 304 | HIGH | 11 | 1 |
| 305 | HIGH | 11 | 2 |
| 306 | HIGH | 11 | 1 |
| 307 | HIGH | 11 | 1 |
| 308 | HIGH | 11 | 1 |
| 309 | HIGH | 11 | 1 |
| 310 | HIGH | 11 | 1 |
| 311 | HIGH | 11 | 1 |
| 312 | HIGH | 11 | 2 |
| 313 | HIGH | 11 | 1 |
| 314 | HIGH | 11 | 1 |
| 315 | HIGH | 11 | 2 |
| 316 | HIGH | 11 | 1 |
| 317 | HIGH | 11 | 3 |
| 318 | HIGH | 11 | 1 |
| 319 | HIGH | 11 | 1 |
| 320 | HIGH | 11 | 2 |
| 321 | HIGH | 11 | 1 |
| 322 | HIGH | 11 | 2 |
| 323 | HIGH | 11 | 1 |
| 324 | HIGH | 11 | 1 |
| 325 | HIGH | 11 | 2 |
| 326 | HIGH | 11 | 2 |
| 327 | HIGH | 11 | 1 |
| 328 | HIGH | 11 | 2 |
| 329 | HIGH | 11 | 1 |
| 330 | HIGH | 11 | 1 |
| 331 | HIGH | 11 | 1 |
| 332 | HIGH | 11 | 1 |
| 333 | HIGH | 11 | 1 |
| 334 | HIGH | 11 | 1 |
| 335 | HIGH | 11 | 1 |
| 336 | HIGH | 11 | 1 |
| 337 | HIGH | 11 | 1 |
| 338 | HIGH | 11 | 1 |
| 339 | HIGH | 11 | 1 |
| 340 | HIGH | 11 | 3 |
| 341 | HIGH | 11 | 1 |
| 342 | HIGH | 11 | 1 |
| 343 | HIGH | 11 | 1 |
| 344 | HIGH | 11 | 1 |
| 345 | HIGH | 11 | 1 |
| 346 | HIGH | 11 | 1 |
| 347 | HIGH | 11 | 1 |
| 348 | HIGH | 11 | 1 |
| 349 | HIGH | 11 | 1 |
| 350 | HIGH | 11 | 1 |
| 351 | HIGH | 11 | 1 |
| 352 | HIGH | 11 | 1 |
| 353 | HIGH | 11 | 1 |
| 354 | HIGH | 11 | 1 |
| 355 | HIGH | 11 | 1 |
| 356 | HIGH | 11 | 1 |
| 357 | MEDIUM | 9 | 1 |
| 358 | MEDIUM | 9 | 1 |
| 359 | MEDIUM | 9 | 1 |
| 360 | MEDIUM | 9 | 1 |
| 361 | MEDIUM | 9 | 1 |
| 362 | MEDIUM | 9 | 1 |
| 363 | MEDIUM | 9 | 1 |
| 364 | MEDIUM | 9 | 1 |
| 365 | MEDIUM | 9 | 1 |
| 366 | MEDIUM | 9 | 2 |
| 367 | MEDIUM | 9 | 1 |
| 368 | MEDIUM | 9 | 1 |
| 369 | MEDIUM | 9 | 1 |
| 370 | MEDIUM | 9 | 1 |
| 371 | MEDIUM | 9 | 1 |
| 372 | MEDIUM | 9 | 1 |
| 373 | MEDIUM | 9 | 1 |
| 374 | MEDIUM | 9 | 1 |
| 375 | MEDIUM | 9 | 1 |
| 376 | MEDIUM | 9 | 1 |
| 377 | MEDIUM | 9 | 1 |
| 378 | MEDIUM | 9 | 1 |
| 379 | MEDIUM | 9 | 1 |
| 380 | MEDIUM | 9 | 1 |
| 381 | MEDIUM | 9 | 1 |
| 382 | MEDIUM | 9 | 1 |
| 383 | MEDIUM | 9 | 1 |
| 384 | MEDIUM | 9 | 1 |
| 385 | MEDIUM | 9 | 1 |
| 386 | MEDIUM | 9 | 1 |
| 387 | MEDIUM | 9 | 1 |
| 388 | MEDIUM | 9 | 1 |
| 389 | MEDIUM | 9 | 1 |
| 390 | MEDIUM | 9 | 1 |
| 391 | MEDIUM | 9 | 1 |
| 392 | MEDIUM | 9 | 1 |
| 393 | MEDIUM | 9 | 1 |
| 394 | MEDIUM | 9 | 1 |
| 395 | MEDIUM | 9 | 1 |
| 396 | MEDIUM | 9 | 1 |
| 397 | MEDIUM | 9 | 1 |
| 398 | MEDIUM | 9 | 1 |
| 399 | MEDIUM | 9 | 1 |
| 400 | MEDIUM | 9 | 1 |
| 401 | MEDIUM | 9 | 1 |
| 402 | MEDIUM | 9 | 1 |
| 403 | MEDIUM | 9 | 1 |
| 404 | MEDIUM | 9 | 2 |
| 405 | MEDIUM | 9 | 1 |
| 406 | MEDIUM | 9 | 1 |
| 407 | MEDIUM | 9 | 2 |
| 408 | MEDIUM | 9 | 1 |
| 409 | MEDIUM | 9 | 1 |
| 410 | MEDIUM | 9 | 1 |
| 411 | MEDIUM | 9 | 1 |
| 412 | MEDIUM | 9 | 1 |
| 413 | MEDIUM | 9 | 1 |
| 414 | MEDIUM | 9 | 1 |
| 415 | MEDIUM | 9 | 1 |
| 416 | MEDIUM | 9 | 1 |
| 417 | MEDIUM | 9 | 1 |
| 418 | MEDIUM | 9 | 1 |
| 419 | MEDIUM | 9 | 1 |
| 420 | MEDIUM | 9 | 1 |
| 421 | MEDIUM | 9 | 1 |
| 422 | MEDIUM | 9 | 1 |
| 423 | MEDIUM | 9 | 1 |
| 424 | MEDIUM | 9 | 1 |
| 425 | MEDIUM | 9 | 1 |
| 426 | MEDIUM | 9 | 1 |
| 427 | MEDIUM | 9 | 1 |
| 428 | MEDIUM | 9 | 1 |
| 429 | MEDIUM | 9 | 1 |
| 430 | MEDIUM | 9 | 1 |
| 431 | MEDIUM | 9 | 1 |
| 432 | MEDIUM | 9 | 1 |
| 433 | MEDIUM | 9 | 1 |
| 434 | MEDIUM | 9 | 1 |
| 435 | MEDIUM | 9 | 2 |
| 436 | MEDIUM | 9 | 1 |
| 437 | MEDIUM | 9 | 1 |
| 438 | MEDIUM | 9 | 1 |
| 439 | MEDIUM | 9 | 1 |
| 440 | MEDIUM | 9 | 1 |
| 441 | MEDIUM | 9 | 1 |
| 442 | MEDIUM | 9 | 1 |
| 443 | MEDIUM | 9 | 3 |
| 444 | MEDIUM | 9 | 1 |
| 445 | MEDIUM | 9 | 1 |
| 446 | MEDIUM | 9 | 1 |
| 447 | MEDIUM | 9 | 1 |
| 448 | MEDIUM | 9 | 1 |
| 449 | MEDIUM | 9 | 1 |
| 450 | MEDIUM | 9 | 1 |
| 451 | MEDIUM | 9 | 1 |
| 452 | MEDIUM | 9 | 1 |
| 453 | MEDIUM | 9 | 1 |
| 454 | MEDIUM | 9 | 2 |
| 455 | MEDIUM | 9 | 1 |
| 456 | MEDIUM | 9 | 1 |
| 457 | MEDIUM | 9 | 1 |
| 458 | MEDIUM | 9 | 1 |
| 459 | MEDIUM | 9 | 1 |
| 460 | MEDIUM | 9 | 1 |
| 461 | MEDIUM | 9 | 3 |
| 462 | MEDIUM | 9 | 1 |
| 463 | MEDIUM | 9 | 1 |
| 464 | MEDIUM | 9 | 1 |
| 465 | MEDIUM | 9 | 2 |
| 466 | MEDIUM | 9 | 1 |
| 467 | MEDIUM | 9 | 1 |
| 468 | MEDIUM | 9 | 1 |
| 469 | MEDIUM | 9 | 1 |
| 470 | MEDIUM | 9 | 2 |
| 471 | MEDIUM | 9 | 1 |
| 472 | MEDIUM | 9 | 1 |
| 473 | MEDIUM | 9 | 1 |
| 474 | MEDIUM | 9 | 1 |
| 475 | MEDIUM | 9 | 1 |
| 476 | MEDIUM | 9 | 1 |
| 477 | MEDIUM | 9 | 2 |
| 478 | MEDIUM | 9 | 1 |
| 479 | MEDIUM | 9 | 2 |
| 480 | MEDIUM | 9 | 1 |
| 481 | MEDIUM | 9 | 2 |
| 482 | MEDIUM | 9 | 1 |
| 483 | MEDIUM | 9 | 1 |
| 484 | MEDIUM | 9 | 1 |
| 485 | MEDIUM | 9 | 1 |
| 486 | MEDIUM | 9 | 1 |
| 487 | MEDIUM | 9 | 1 |
| 488 | MEDIUM | 9 | 1 |
| 489 | MEDIUM | 9 | 1 |
| 490 | MEDIUM | 9 | 1 |
| 491 | MEDIUM | 9 | 1 |
| 492 | MEDIUM | 9 | 1 |
| 493 | MEDIUM | 9 | 2 |
| 494 | MEDIUM | 9 | 1 |
| 495 | MEDIUM | 9 | 1 |
| 496 | MEDIUM | 9 | 2 |
| 497 | MEDIUM | 9 | 1 |
| 498 | MEDIUM | 9 | 1 |
| 499 | MEDIUM | 9 | 1 |
| 500 | MEDIUM | 9 | 1 |
| 501 | MEDIUM | 9 | 1 |
| 502 | MEDIUM | 9 | 2 |
| 503 | MEDIUM | 9 | 1 |
| 504 | MEDIUM | 9 | 2 |
| 505 | MEDIUM | 9 | 1 |
| 506 | MEDIUM | 9 | 1 |
| 507 | MEDIUM | 9 | 1 |
| 508 | MEDIUM | 9 | 1 |
| 509 | MEDIUM | 9 | 1 |
| 510 | MEDIUM | 9 | 1 |
| 511 | MEDIUM | 9 | 1 |
| 512 | MEDIUM | 9 | 1 |
| 513 | MEDIUM | 9 | 1 |
| 514 | MEDIUM | 9 | 1 |
| 515 | MEDIUM | 9 | 2 |
| 516 | MEDIUM | 9 | 1 |
| 517 | MEDIUM | 9 | 2 |
| 518 | MEDIUM | 9 | 1 |
| 519 | MEDIUM | 9 | 1 |
| 520 | MEDIUM | 9 | 1 |
| 521 | MEDIUM | 9 | 2 |
| 522 | MEDIUM | 9 | 1 |
| 523 | MEDIUM | 9 | 2 |
| 524 | MEDIUM | 9 | 1 |
| 525 | MEDIUM | 9 | 1 |
| 526 | MEDIUM | 9 | 1 |
| 527 | MEDIUM | 9 | 1 |
| 528 | MEDIUM | 9 | 2 |
| 529 | MEDIUM | 9 | 2 |
| 530 | MEDIUM | 9 | 1 |
| 531 | MEDIUM | 9 | 1 |
| 532 | MEDIUM | 9 | 1 |
| 533 | MEDIUM | 9 | 1 |
| 534 | MEDIUM | 9 | 1 |
| 535 | MEDIUM | 9 | 1 |
| 536 | MEDIUM | 9 | 1 |
| 537 | MEDIUM | 9 | 1 |
| 538 | MEDIUM | 9 | 1 |
| 539 | MEDIUM | 9 | 1 |
| 540 | MEDIUM | 9 | 1 |
| 541 | MEDIUM | 9 | 1 |
| 542 | MEDIUM | 9 | 1 |
| 543 | MEDIUM | 9 | 1 |
| 544 | MEDIUM | 9 | 1 |
| 545 | MEDIUM | 9 | 1 |
| 546 | MEDIUM | 9 | 1 |
| 547 | MEDIUM | 9 | 2 |
| 548 | MEDIUM | 9 | 1 |
| 549 | MEDIUM | 9 | 1 |
| 550 | MEDIUM | 9 | 1 |
| 551 | MEDIUM | 9 | 2 |
| 552 | MEDIUM | 9 | 1 |
| 553 | MEDIUM | 9 | 1 |
| 554 | MEDIUM | 9 | 1 |
| 555 | MEDIUM | 9 | 1 |
| 556 | MEDIUM | 9 | 1 |
| 557 | MEDIUM | 9 | 1 |
| 558 | MEDIUM | 9 | 1 |
| 559 | MEDIUM | 9 | 1 |
| 560 | MEDIUM | 9 | 1 |
| 561 | MEDIUM | 9 | 1 |
| 562 | MEDIUM | 9 | 1 |
| 563 | MEDIUM | 9 | 1 |
| 564 | MEDIUM | 9 | 1 |
| 565 | MEDIUM | 9 | 1 |
| 566 | MEDIUM | 9 | 1 |
| 567 | MEDIUM | 9 | 1 |
| 568 | MEDIUM | 9 | 1 |
| 569 | MEDIUM | 9 | 1 |
| 570 | MEDIUM | 9 | 1 |
| 571 | MEDIUM | 9 | 1 |
| 572 | MEDIUM | 9 | 1 |
| 573 | MEDIUM | 9 | 1 |
| 574 | MEDIUM | 9 | 1 |
| 575 | MEDIUM | 9 | 2 |
| 576 | MEDIUM | 9 | 1 |
| 577 | MEDIUM | 9 | 1 |
| 578 | MEDIUM | 9 | 1 |
| 579 | MEDIUM | 9 | 1 |
| 580 | MEDIUM | 9 | 1 |
| 581 | MEDIUM | 9 | 1 |
| 582 | MEDIUM | 9 | 2 |
| 583 | MEDIUM | 9 | 2 |
| 584 | MEDIUM | 9 | 1 |
| 585 | MEDIUM | 9 | 2 |
| 586 | MEDIUM | 9 | 1 |
| 587 | MEDIUM | 9 | 2 |
| 588 | MEDIUM | 9 | 1 |
| 589 | MEDIUM | 9 | 1 |
| 590 | MEDIUM | 9 | 1 |
| 591 | MEDIUM | 9 | 1 |
| 592 | MEDIUM | 9 | 1 |
| 593 | MEDIUM | 9 | 1 |
| 594 | MEDIUM | 9 | 1 |
| 595 | MEDIUM | 9 | 1 |
| 596 | MEDIUM | 9 | 1 |
| 597 | MEDIUM | 9 | 1 |
| 598 | MEDIUM | 9 | 1 |
| 599 | MEDIUM | 9 | 1 |
| 600 | MEDIUM | 9 | 1 |
| 601 | MEDIUM | 9 | 1 |
| 602 | MEDIUM | 8 | 1 |
| 603 | MEDIUM | 8 | 1 |
| 604 | MEDIUM | 8 | 1 |
| 605 | MEDIUM | 8 | 1 |
| 606 | MEDIUM | 8 | 1 |
| 607 | MEDIUM | 8 | 1 |
| 608 | MEDIUM | 8 | 1 |
| 609 | MEDIUM | 8 | 1 |
| 610 | MEDIUM | 8 | 1 |
| 611 | MEDIUM | 8 | 1 |
| 612 | MEDIUM | 8 | 1 |
| 613 | MEDIUM | 8 | 1 |
| 614 | MEDIUM | 8 | 1 |
| 615 | MEDIUM | 8 | 1 |
| 616 | MEDIUM | 8 | 1 |
| 617 | MEDIUM | 8 | 1 |
| 618 | MEDIUM | 8 | 1 |
| 619 | MEDIUM | 8 | 1 |
| 620 | MEDIUM | 8 | 1 |
| 621 | MEDIUM | 8 | 1 |
| 622 | MEDIUM | 8 | 1 |
| 623 | MEDIUM | 8 | 1 |
| 624 | MEDIUM | 8 | 1 |
| 625 | MEDIUM | 8 | 2 |
| 626 | MEDIUM | 8 | 2 |
| 627 | MEDIUM | 8 | 1 |
| 628 | MEDIUM | 8 | 2 |
| 629 | MEDIUM | 8 | 1 |
| 630 | MEDIUM | 8 | 1 |
| 631 | MEDIUM | 8 | 1 |
| 632 | MEDIUM | 8 | 1 |
| 633 | MEDIUM | 8 | 1 |
| 634 | MEDIUM | 8 | 1 |
| 635 | MEDIUM | 8 | 1 |
| 636 | MEDIUM | 8 | 1 |
| 637 | MEDIUM | 8 | 1 |
| 638 | MEDIUM | 8 | 1 |
| 639 | MEDIUM | 8 | 1 |
| 640 | MEDIUM | 8 | 1 |
| 641 | MEDIUM | 8 | 1 |
| 642 | MEDIUM | 8 | 1 |
| 643 | MEDIUM | 8 | 1 |
| 644 | MEDIUM | 8 | 1 |
| 645 | MEDIUM | 8 | 1 |
| 646 | MEDIUM | 8 | 1 |
| 647 | MEDIUM | 8 | 1 |
| 648 | MEDIUM | 8 | 1 |
| 649 | MEDIUM | 8 | 1 |
| 650 | MEDIUM | 8 | 1 |
| 651 | MEDIUM | 8 | 1 |
| 652 | MEDIUM | 8 | 1 |
| 653 | MEDIUM | 8 | 1 |
| 654 | MEDIUM | 8 | 1 |
| 655 | MEDIUM | 8 | 1 |
| 656 | MEDIUM | 8 | 2 |
| 657 | MEDIUM | 8 | 1 |
| 658 | MEDIUM | 8 | 1 |
| 659 | MEDIUM | 8 | 1 |
| 660 | MEDIUM | 8 | 1 |
| 661 | MEDIUM | 8 | 1 |
| 662 | MEDIUM | 8 | 1 |
| 663 | MEDIUM | 8 | 1 |
| 664 | MEDIUM | 8 | 1 |
| 665 | MEDIUM | 8 | 1 |
| 666 | MEDIUM | 8 | 1 |
| 667 | MEDIUM | 8 | 1 |
| 668 | MEDIUM | 8 | 1 |
| 669 | MEDIUM | 8 | 1 |
| 670 | MEDIUM | 8 | 1 |
| 671 | MEDIUM | 8 | 1 |
| 672 | MEDIUM | 8 | 1 |
| 673 | MEDIUM | 8 | 1 |
| 674 | MEDIUM | 8 | 1 |
| 675 | MEDIUM | 8 | 1 |
| 676 | MEDIUM | 8 | 1 |
| 677 | MEDIUM | 8 | 1 |
| 678 | MEDIUM | 8 | 1 |
| 679 | MEDIUM | 8 | 1 |
| 680 | MEDIUM | 8 | 1 |
| 681 | MEDIUM | 8 | 1 |
| 682 | MEDIUM | 8 | 1 |
| 683 | MEDIUM | 8 | 1 |
| 684 | MEDIUM | 8 | 1 |
| 685 | MEDIUM | 8 | 1 |
| 686 | MEDIUM | 8 | 1 |
| 687 | MEDIUM | 8 | 2 |
| 688 | MEDIUM | 8 | 1 |
| 689 | MEDIUM | 8 | 1 |
| 690 | MEDIUM | 8 | 1 |
| 691 | MEDIUM | 8 | 1 |
| 692 | MEDIUM | 8 | 1 |
| 693 | MEDIUM | 8 | 1 |
| 694 | MEDIUM | 8 | 2 |
| 695 | MEDIUM | 8 | 1 |
| 696 | MEDIUM | 8 | 1 |
| 697 | MEDIUM | 8 | 1 |
| 698 | MEDIUM | 8 | 1 |
| 699 | MEDIUM | 8 | 1 |
| 700 | MEDIUM | 8 | 1 |
| 701 | MEDIUM | 8 | 1 |
| 702 | MEDIUM | 8 | 1 |
| 703 | MEDIUM | 8 | 1 |
| 704 | MEDIUM | 8 | 1 |
| 705 | MEDIUM | 8 | 1 |
| 706 | MEDIUM | 8 | 1 |
| 707 | MEDIUM | 8 | 1 |
| 708 | MEDIUM | 8 | 2 |
| 709 | MEDIUM | 8 | 1 |
| 710 | MEDIUM | 8 | 1 |
| 711 | MEDIUM | 8 | 2 |
| 712 | MEDIUM | 8 | 1 |
| 713 | MEDIUM | 8 | 1 |
| 714 | MEDIUM | 8 | 1 |
| 715 | MEDIUM | 8 | 1 |
| 716 | MEDIUM | 8 | 1 |
| 717 | MEDIUM | 8 | 1 |
| 718 | MEDIUM | 8 | 1 |
| 719 | MEDIUM | 8 | 1 |
| 720 | MEDIUM | 8 | 1 |
| 721 | MEDIUM | 8 | 1 |
| 722 | MEDIUM | 8 | 1 |
| 723 | MEDIUM | 8 | 1 |
| 724 | MEDIUM | 8 | 1 |
| 725 | MEDIUM | 8 | 1 |
| 726 | MEDIUM | 8 | 1 |
| 727 | MEDIUM | 8 | 1 |
| 728 | MEDIUM | 8 | 1 |
| 729 | MEDIUM | 8 | 1 |
| 730 | MEDIUM | 8 | 1 |
| 731 | MEDIUM | 8 | 1 |
| 732 | MEDIUM | 8 | 1 |
| 733 | MEDIUM | 8 | 1 |
| 734 | MEDIUM | 8 | 1 |
| 735 | MEDIUM | 8 | 1 |
| 736 | MEDIUM | 8 | 1 |
| 737 | MEDIUM | 8 | 1 |
| 738 | MEDIUM | 8 | 1 |
| 739 | MEDIUM | 8 | 1 |
| 740 | MEDIUM | 8 | 1 |
| 741 | MEDIUM | 8 | 1 |
| 742 | MEDIUM | 8 | 1 |
| 743 | MEDIUM | 8 | 1 |
| 744 | MEDIUM | 8 | 1 |
| 745 | MEDIUM | 8 | 1 |
| 746 | MEDIUM | 8 | 1 |
| 747 | MEDIUM | 8 | 1 |
| 748 | MEDIUM | 8 | 1 |
| 749 | MEDIUM | 8 | 1 |
| 750 | MEDIUM | 8 | 1 |
| 751 | MEDIUM | 8 | 3 |
| 752 | MEDIUM | 8 | 1 |
| 753 | MEDIUM | 8 | 1 |
| 754 | MEDIUM | 8 | 2 |
| 755 | MEDIUM | 8 | 1 |
| 756 | MEDIUM | 8 | 1 |
| 757 | MEDIUM | 8 | 1 |
| 758 | MEDIUM | 8 | 1 |
| 759 | MEDIUM | 8 | 2 |
| 760 | MEDIUM | 8 | 1 |
| 761 | MEDIUM | 8 | 1 |
| 762 | MEDIUM | 8 | 1 |
| 763 | MEDIUM | 8 | 1 |
| 764 | MEDIUM | 8 | 1 |
| 765 | MEDIUM | 8 | 1 |
| 766 | MEDIUM | 8 | 1 |
| 767 | MEDIUM | 8 | 1 |
| 768 | MEDIUM | 8 | 1 |
| 769 | MEDIUM | 8 | 1 |
| 770 | MEDIUM | 8 | 1 |
| 771 | MEDIUM | 8 | 1 |
| 772 | MEDIUM | 8 | 1 |
| 773 | MEDIUM | 8 | 1 |
| 774 | MEDIUM | 8 | 1 |
| 775 | MEDIUM | 8 | 1 |
| 776 | MEDIUM | 8 | 1 |
| 777 | MEDIUM | 8 | 1 |
| 778 | MEDIUM | 8 | 1 |
| 779 | MEDIUM | 8 | 1 |
| 780 | MEDIUM | 8 | 1 |
| 781 | MEDIUM | 8 | 1 |
| 782 | MEDIUM | 8 | 2 |
| 783 | MEDIUM | 8 | 1 |
| 784 | MEDIUM | 8 | 1 |
| 785 | MEDIUM | 8 | 1 |
| 786 | MEDIUM | 8 | 1 |
| 787 | MEDIUM | 8 | 1 |
| 788 | MEDIUM | 8 | 1 |
| 789 | MEDIUM | 8 | 1 |
| 790 | MEDIUM | 8 | 1 |
| 791 | MEDIUM | 8 | 1 |
| 792 | MEDIUM | 8 | 1 |
| 793 | MEDIUM | 8 | 1 |
| 794 | MEDIUM | 8 | 1 |
| 795 | MEDIUM | 8 | 1 |
| 796 | MEDIUM | 8 | 1 |
| 797 | MEDIUM | 8 | 3 |
| 798 | MEDIUM | 8 | 1 |
| 799 | MEDIUM | 8 | 1 |
| 800 | MEDIUM | 8 | 1 |
| 801 | MEDIUM | 8 | 1 |
| 802 | MEDIUM | 8 | 1 |
| 803 | MEDIUM | 8 | 1 |
| 804 | MEDIUM | 8 | 1 |
| 805 | MEDIUM | 8 | 1 |
| 806 | MEDIUM | 8 | 1 |
| 807 | MEDIUM | 8 | 1 |
| 808 | MEDIUM | 8 | 1 |
| 809 | MEDIUM | 8 | 2 |
| 810 | MEDIUM | 8 | 1 |
| 811 | MEDIUM | 8 | 1 |
| 812 | MEDIUM | 8 | 1 |
| 813 | MEDIUM | 8 | 1 |
| 814 | MEDIUM | 8 | 1 |
| 815 | MEDIUM | 8 | 1 |
| 816 | MEDIUM | 8 | 1 |
| 817 | MEDIUM | 8 | 1 |
| 818 | MEDIUM | 8 | 1 |
| 819 | MEDIUM | 8 | 1 |
| 820 | MEDIUM | 8 | 1 |
| 821 | MEDIUM | 8 | 1 |
| 822 | MEDIUM | 8 | 1 |
| 823 | MEDIUM | 8 | 1 |
| 824 | MEDIUM | 8 | 1 |
| 825 | MEDIUM | 8 | 1 |
| 826 | MEDIUM | 8 | 1 |
| 827 | MEDIUM | 8 | 1 |
| 828 | MEDIUM | 8 | 1 |
| 829 | MEDIUM | 8 | 1 |
| 830 | MEDIUM | 8 | 1 |
| 831 | MEDIUM | 8 | 1 |
| 832 | MEDIUM | 8 | 1 |
| 833 | MEDIUM | 8 | 1 |
| 834 | MEDIUM | 8 | 1 |
| 835 | MEDIUM | 8 | 1 |
| 836 | MEDIUM | 8 | 1 |
| 837 | MEDIUM | 8 | 1 |
| 838 | MEDIUM | 8 | 1 |
| 839 | MEDIUM | 8 | 1 |
| 840 | MEDIUM | 8 | 1 |
| 841 | MEDIUM | 8 | 1 |
| 842 | MEDIUM | 8 | 1 |
| 843 | MEDIUM | 8 | 1 |
| 844 | MEDIUM | 8 | 1 |
| 845 | MEDIUM | 8 | 1 |
| 846 | MEDIUM | 8 | 1 |
| 847 | MEDIUM | 8 | 1 |
| 848 | MEDIUM | 8 | 1 |
| 849 | MEDIUM | 8 | 1 |
| 850 | MEDIUM | 8 | 1 |
| 851 | MEDIUM | 8 | 1 |
| 852 | MEDIUM | 8 | 1 |
| 853 | MEDIUM | 8 | 1 |
| 854 | MEDIUM | 8 | 1 |
| 855 | MEDIUM | 8 | 1 |
| 856 | MEDIUM | 8 | 1 |
| 857 | MEDIUM | 8 | 1 |
| 858 | MEDIUM | 8 | 1 |
| 859 | MEDIUM | 8 | 1 |
| 860 | MEDIUM | 8 | 1 |
| 861 | MEDIUM | 8 | 2 |
| 862 | MEDIUM | 8 | 1 |
| 863 | MEDIUM | 8 | 2 |
| 864 | MEDIUM | 8 | 1 |
| 865 | MEDIUM | 8 | 1 |
| 866 | MEDIUM | 8 | 1 |
| 867 | MEDIUM | 8 | 1 |
| 868 | MEDIUM | 8 | 1 |
| 869 | MEDIUM | 8 | 1 |
| 870 | MEDIUM | 8 | 1 |
| 871 | MEDIUM | 8 | 1 |
| 872 | MEDIUM | 8 | 2 |
| 873 | MEDIUM | 8 | 1 |
| 874 | MEDIUM | 8 | 1 |
| 875 | MEDIUM | 8 | 1 |
| 876 | MEDIUM | 8 | 1 |
| 877 | MEDIUM | 8 | 1 |
| 878 | MEDIUM | 8 | 1 |
| 879 | MEDIUM | 8 | 1 |
| 880 | MEDIUM | 8 | 1 |
| 881 | MEDIUM | 8 | 1 |
| 882 | MEDIUM | 8 | 2 |
| 883 | MEDIUM | 8 | 2 |
| 884 | MEDIUM | 8 | 1 |
| 885 | MEDIUM | 8 | 1 |
| 886 | MEDIUM | 8 | 1 |
| 887 | MEDIUM | 8 | 1 |
| 888 | MEDIUM | 8 | 1 |
| 889 | MEDIUM | 8 | 1 |
| 890 | MEDIUM | 8 | 2 |
| 891 | MEDIUM | 8 | 2 |
| 892 | MEDIUM | 8 | 1 |
| 893 | MEDIUM | 8 | 1 |
| 894 | MEDIUM | 8 | 1 |
| 895 | MEDIUM | 8 | 1 |
| 896 | MEDIUM | 8 | 1 |
| 897 | MEDIUM | 8 | 1 |
| 898 | MEDIUM | 8 | 1 |
| 899 | MEDIUM | 8 | 1 |
| 900 | MEDIUM | 8 | 1 |
| 901 | MEDIUM | 8 | 1 |
| 902 | MEDIUM | 8 | 1 |
| 903 | MEDIUM | 8 | 1 |
| 904 | MEDIUM | 8 | 1 |
| 905 | MEDIUM | 8 | 1 |
| 906 | MEDIUM | 8 | 1 |
| 907 | MEDIUM | 8 | 1 |
| 908 | MEDIUM | 8 | 1 |
| 909 | MEDIUM | 8 | 1 |
| 910 | MEDIUM | 8 | 1 |
| 911 | MEDIUM | 8 | 1 |
| 912 | MEDIUM | 8 | 1 |
| 913 | MEDIUM | 8 | 1 |
| 914 | MEDIUM | 8 | 1 |
| 915 | MEDIUM | 8 | 1 |
| 916 | MEDIUM | 8 | 1 |
| 917 | MEDIUM | 10 | 2 |
| 918 | MEDIUM | 10 | 1 |
| 919 | MEDIUM | 10 | 1 |
| 920 | MEDIUM | 10 | 1 |
| 921 | MEDIUM | 10 | 1 |
| 922 | MEDIUM | 10 | 1 |
| 923 | MEDIUM | 10 | 1 |
| 924 | MEDIUM | 10 | 1 |
| 925 | MEDIUM | 10 | 1 |
| 926 | MEDIUM | 10 | 1 |
| 927 | MEDIUM | 10 | 1 |
| 928 | MEDIUM | 10 | 2 |
| 929 | MEDIUM | 10 | 1 |
| 930 | MEDIUM | 10 | 1 |
| 931 | MEDIUM | 10 | 1 |
| 932 | MEDIUM | 10 | 1 |
| 933 | MEDIUM | 10 | 2 |
| 934 | MEDIUM | 10 | 2 |
| 935 | MEDIUM | 10 | 1 |
| 936 | MEDIUM | 10 | 1 |
| 937 | MEDIUM | 10 | 1 |
| 938 | MEDIUM | 10 | 2 |
| 939 | MEDIUM | 10 | 1 |
| 940 | MEDIUM | 10 | 1 |
| 941 | MEDIUM | 10 | 2 |
| 942 | MEDIUM | 10 | 1 |
| 943 | MEDIUM | 10 | 1 |
| 944 | MEDIUM | 10 | 1 |
| 945 | MEDIUM | 10 | 1 |
| 946 | MEDIUM | 10 | 1 |
| 947 | MEDIUM | 10 | 1 |
| 948 | MEDIUM | 10 | 1 |
| 949 | MEDIUM | 10 | 2 |
| 950 | MEDIUM | 10 | 1 |
| 951 | MEDIUM | 10 | 1 |
| 952 | MEDIUM | 10 | 1 |
| 953 | MEDIUM | 10 | 1 |
| 954 | MEDIUM | 10 | 2 |
| 955 | MEDIUM | 10 | 1 |
| 956 | MEDIUM | 10 | 1 |
| 957 | MEDIUM | 10 | 1 |
| 958 | MEDIUM | 10 | 2 |
| 959 | MEDIUM | 10 | 1 |
| 960 | MEDIUM | 10 | 1 |
| 961 | MEDIUM | 10 | 1 |
| 962 | MEDIUM | 10 | 1 |
| 963 | MEDIUM | 10 | 2 |
| 964 | MEDIUM | 10 | 3 |
| 965 | MEDIUM | 10 | 1 |
| 966 | MEDIUM | 10 | 1 |
| 967 | MEDIUM | 10 | 2 |
| 968 | MEDIUM | 10 | 1 |
| 969 | MEDIUM | 10 | 2 |
| 970 | MEDIUM | 10 | 1 |
| 971 | MEDIUM | 10 | 1 |
| 972 | MEDIUM | 10 | 1 |
| 973 | MEDIUM | 10 | 2 |
| 974 | MEDIUM | 10 | 2 |
| 975 | MEDIUM | 10 | 1 |
| 976 | MEDIUM | 10 | 1 |
| 977 | MEDIUM | 10 | 1 |
| 978 | MEDIUM | 10 | 1 |
| 979 | MEDIUM | 10 | 1 |
| 980 | MEDIUM | 10 | 1 |
| 981 | MEDIUM | 10 | 1 |
| 982 | MEDIUM | 10 | 1 |
| 983 | MEDIUM | 10 | 1 |
| 984 | MEDIUM | 10 | 1 |
| 985 | MEDIUM | 10 | 1 |
| 986 | MEDIUM | 10 | 1 |
| 987 | MEDIUM | 10 | 1 |
| 988 | MEDIUM | 10 | 1 |
| 989 | MEDIUM | 10 | 1 |
| 990 | MEDIUM | 10 | 2 |
| 991 | MEDIUM | 10 | 2 |
| 992 | MEDIUM | 10 | 2 |
| 993 | MEDIUM | 10 | 1 |
| 994 | MEDIUM | 10 | 1 |
| 995 | MEDIUM | 10 | 1 |
| 996 | MEDIUM | 10 | 1 |
| 997 | MEDIUM | 10 | 1 |
| 998 | MEDIUM | 10 | 1 |
| 999 | MEDIUM | 10 | 1 |
| 1000 | MEDIUM | 10 | 1 |
| 1001 | MEDIUM | 10 | 1 |
| 1002 | MEDIUM | 10 | 2 |
| 1003 | MEDIUM | 10 | 2 |
| 1004 | MEDIUM | 10 | 1 |
| 1005 | MEDIUM | 10 | 1 |
| 1006 | MEDIUM | 10 | 1 |
| 1007 | MEDIUM | 10 | 1 |
| 1008 | MEDIUM | 10 | 1 |
| 1009 | MEDIUM | 10 | 1 |
| 1010 | MEDIUM | 10 | 2 |
| 1011 | MEDIUM | 10 | 1 |
| 1012 | MEDIUM | 10 | 1 |
| 1013 | MEDIUM | 10 | 1 |
| 1014 | MEDIUM | 10 | 1 |
| 1015 | MEDIUM | 10 | 1 |
| 1016 | MEDIUM | 10 | 1 |
| 1017 | MEDIUM | 10 | 2 |
| 1018 | MEDIUM | 10 | 2 |
| 1019 | MEDIUM | 10 | 2 |
| 1020 | MEDIUM | 10 | 1 |
| 1021 | MEDIUM | 10 | 1 |
| 1022 | MEDIUM | 10 | 1 |
| 1023 | MEDIUM | 10 | 1 |
| 1024 | MEDIUM | 10 | 1 |
| 1025 | MEDIUM | 10 | 1 |
| 1026 | MEDIUM | 10 | 1 |
| 1027 | MEDIUM | 10 | 1 |
| 1028 | MEDIUM | 10 | 1 |
| 1029 | MEDIUM | 10 | 1 |
| 1030 | MEDIUM | 10 | 2 |
| 1031 | MEDIUM | 10 | 2 |
| 1032 | MEDIUM | 10 | 1 |
| 1033 | MEDIUM | 10 | 2 |
| 1034 | MEDIUM | 10 | 1 |
| 1035 | MEDIUM | 10 | 1 |
| 1036 | MEDIUM | 10 | 1 |
| 1037 | MEDIUM | 10 | 1 |
| 1038 | MEDIUM | 10 | 2 |
| 1039 | MEDIUM | 10 | 1 |
| 1040 | MEDIUM | 10 | 1 |
| 1041 | MEDIUM | 10 | 1 |
| 1042 | MEDIUM | 10 | 1 |
| 1043 | MEDIUM | 10 | 1 |
| 1044 | MEDIUM | 10 | 2 |
| 1045 | MEDIUM | 10 | 2 |
| 1046 | MEDIUM | 10 | 1 |
| 1047 | MEDIUM | 10 | 2 |
| 1048 | MEDIUM | 10 | 1 |
| 1049 | MEDIUM | 10 | 1 |
| 1050 | MEDIUM | 10 | 1 |
| 1051 | MEDIUM | 10 | 1 |
| 1052 | MEDIUM | 10 | 2 |
| 1053 | MEDIUM | 10 | 2 |
| 1054 | MEDIUM | 10 | 1 |
| 1055 | MEDIUM | 10 | 1 |
| 1056 | MEDIUM | 10 | 1 |
| 1057 | MEDIUM | 10 | 2 |
| 1058 | MEDIUM | 10 | 2 |
| 1059 | MEDIUM | 10 | 1 |
| 1060 | MEDIUM | 10 | 1 |
| 1061 | MEDIUM | 10 | 1 |
| 1062 | MEDIUM | 10 | 1 |
| 1063 | MEDIUM | 10 | 1 |
| 1064 | MEDIUM | 10 | 1 |
| 1065 | MEDIUM | 10 | 1 |
| 1066 | MEDIUM | 10 | 3 |
| 1067 | MEDIUM | 10 | 2 |
| 1068 | MEDIUM | 10 | 1 |
