# Supplementary material for: Calculation of a Primary Immunodeficiency “Risk Vital Sign” via Population-Wide Analysis of Claims Data to Aid in Clinical Decision Support
Source: Front Pediatr. 2019 Mar 18;7:70. doi: 10.3389/fped.2019.00070 (PMC6431644; doi:10.3389/fped.2019.00070)
Supplement: Supplementary file 3 [file Table_3.DOCX]

| **Supplemental Table 3: Diagnostic Category found in PI-MHR but not in Non-PI MHR Cohorts** | |
| --- | --- |
|  |  |
| **ICD Code (3 Digit)** | **Description** |
| A85 | Other viral encephalitis, not elsewhere classified |
| A89 | Unspecified viral infection of central nervous system |
| B25 | Cytomegaloviral disease |
| B44 | Aspergillosis |
| D82 | Immunodeficiency associated with other major defects |
| D83 | Common variable immunodeficiency |
| D84 | Other immunodeficiencies |
| E13 | Other specified diabetes mellitus |
| F23 | Brief psychotic disorder |
| H33 | Retinal detachments and breaks |
| I11 | Hypertensive heart disease |
| I30 | Acute pericarditis |
| I96 | Gangrene, not elsewhere classified |
| J47 | Bronchiectasis |
| J85 | Abscess of lung and mediastinum |
| K04 | Diseases of pulp and periapical tissues |
| K57 | Diverticular disease of intestine |
| L56 | Other acute skin changes due to ultraviolet radiation |
| M16 | Osteoarthritis of hip |
| M51 | Thoracic, thoracolumbar, and lumbosacral intervertebral disc disorders |
| N17 | Acute kidney failure |
| N18 | Chronic kidney disease (CKD) |
| Q13 | Congenital malformations of anterior segment of eye |
| R65 | Symptoms and signs specifically associated with systemic inflammation and infection |
| T38 | Poisoning by, adverse effect of and underdosing of hormones and their synthetic substitutes and antagonists, not elsewhere classified |
| T86 | Complications of transplanted organs and tissue |
| Z94 | Transplanted organ and tissue status |
